# Experimental analysis and genome mining for functional validation of genes associated with anti-inflammatory, antioxidant, and antibacterial activities in *Kurthia gibsonii* VITAM20

**DOI:** 10.3389/fphar.2026.1799206

**Published:** 2026-07-03

**Authors:** Anju Kanjirakandi Ashokan, Mohanasrinivasan Vaithilingam

**Affiliations:** School of Bio Sciences and Technology, Vellore Institute of Technology, Vellore, Tamil Nadu, India

**Keywords:** anti-inflammatory protease, bioactive molecule, inflammation, *Kurthia gibsonii*, secondary metabolites

## Abstract

**Introduction:**

The identification of microbial strains harbouring bioactive genes is important for the discovery of novel therapeutic agents. The study objective was to identify the genes in *Kurthia gibsonii* VITAM20 that are associated with anti-inflammatory and antioxidant properties and validate these findings with *in vitro* functional assays.

**Methods:**

Genes associated with anti-inflammatory and antioxidant activities were identified by BLAST analysis against curated databases. The anti-inflammatory, antioxidant, and antibacterial potential of *Kurthia gibsonii* VITAM20 were subsequently evaluated using *in vitro* assays.

**Results:**

BLAST analysis identified several genes associated with anti‐inflammatory properties, including *eps* (95.455%), *NEMO* (100%), *spa* (100%), and *spaB* (90.625%). Multiple genes associated with antioxidant potential were also detected, including *CAT* (92.308%), *dps* (95.238%), *gshR3* (91.304%), *gshR4* (100%), *KATA* (100%), *oxyR* (100%), *KatA* (88.462%), *perR* (74.041%, bit score 126), *sodA* (100%), *sodM* (76.471%), and *trxA* (77.778%). The genomic findings were supported by *in vitro* analyses, which demonstrated notable anti‐inflammatory activity. *Kurthia gibsonii* VITAM20 exhibited human red blood cell membrane stabilization rates of 90.78% ± 1.03% and 69.92% under non-heated and heated conditions, respectively, compared with diclofenac (83.01% ± 0.72% and 83.83% ± 1.23%). In the egg albumin denaturation assay, *Kuthia gibsonii* VITAM20 demonstrated a concentration‐dependent increase in inhibition from 80.94% ± 0.57% to 84.47% ± 0.39%, comparable to that of diclofenac. Furthermore, *Kurthia gibsonii* VITAM20 exhibited 64.27% ± 0.46% antioxidant activity compared with ascorbic acid (91.40% ± 0.46%), with an IC50 value of 286.5 µg/mL. The crude enzyme extract demonstrated antibacterial activity against *Listeria monocytogenes* and *Staphylococcus aureus*, producing inhibition zones of 13 mm. The MIC was determined to be 250 µg/mL for both pathogens. At this concentration, the crude enzyme extract inhibited the growth of *L. monocytogenes* and *S. aureus* by 81.46% and 77.82%, respectively.

**Discussion:**

The combined genomic and functional analyses confirmed the presence of conserved genes associated with anti‐inflammatory and antioxidant activities in *Kurthia gibsonii* VITAM20. These findings suggest that *Kurthia gibsonii* VITAM20 represents a promising source of bioactive molecules with potential therapeutic applications.

## 1 Introduction

Inflammation is a key biological response to injury or stimuli, such as the entry of pathogens (Gusev and Zhuravleva, 2022). This process involves vasodilation, increased vascular permeability, and plasma leakage, which causes pain, elevated body temperature, and swelling (Melnikova et al., 2018). Traditional anti-inflammatory remedies have been widely used in the treatment of inflammatory conditions (Ali et al., 2023). However, these drugs usually cause side effects, including digestive problems and kidney failure, while their continued usage leads to the increase of reduced therapeutic effectiveness and drug resistance (Sohail et al., 2023). In response to the existing limitations, enzyme-based treatments have shifted toward varied approaches, such as high specificity, catalytic efficacy, and reduced adverse effects.

Enzymes produced by bacteria exhibit increased bioactivity, including anti-inflammatory, antioxidant, and antibacterial properties. These enzymes are effective against inflammation through regulating the inflammatory signaling pathways and inhibiting the production of pro-inflammatory agents (Liu et al., 2022). The antioxidant properties of these enzymes are involved in the absorption of reactive oxygen species (ROS) and the chelation of metal ions, which are significant for reducing oxidative stress (Tumilaar et al., 2024). In response to the existing limitations, enzyme-based treatments have shifted toward varied approaches, such as high specificity, catalytic efficacy, and reduced adverse effects.

Additionally, these bacterial enzymes demonstrated a notable antibacterial effect by degrading the walls of microbial cells and interrupting biofilm development, which consequently decreased the spread and strength of disease-causing microorganisms. The role of inflammation is significant in host defense and tissue repair, yet, when there is an imbalance, it can often lead to different acute and chronic diseases, encompassing autoimmune conditions, tumors, metabolic issues, and heart-related problems. The current study focused on a bacterium named *Kurthia gibsonii* VITAM20 isolated from butcher soil samples collected in Vellore, Tamil Nadu. The antioxidant, antimicrobial, and anti-inflammatory properties of this isolate were systematically evaluated by genome mining and confirmed by *in vitro* assays. This approach aimed to find novel bioactive enzymes with potential therapeutic and industrial applications.

## 2 Materials and methods

### 2.1 Isolation and identification of the bacteria

Soil samples were taken from Viruthampet (12.94152°N, 79.1351°E), Vellore, Tamil Nadu, India. Serial dilutions were performed, and aliquots were spread on agar plates. The plates were kept at 37 °C for 48 h. After incubation, plates showing different microbial colonies were chosen for further studies. A potent bacterial isolate named VITAM20 was identified through morphological, biochemical, and whole-genome sequencing (Chauhan and Samant, 2022). (The datasets generated and analyzed during the current study are available under the name Mohanasrinivasan V repository; Submission ID: SUB15186439, BioProject ID: PRJNA1238542, BioSample: SAMN47475936, SRA: SRR35894991, Organism: *Kurthia gibsonii* VITAM20; https://www.ncbi.nlm.nih.gov/sra/PRJNA1238542.)

### 2.2 Determination of the growth curve

A single bacterial colony was inoculated into fresh liquid nutrient broth medium and incubated overnight at 30 °C and 250 rpm until the culture reached a stationary phase. After that, 1 mL of the inoculum was transferred to 100 mL of nutrient broth and incubated at 30 °C and 250 rpm for 48 h, along with setting up a control. Optical density at 600 nm was measured at every 2-h intervals (Roy et al., 2025).

### 2.3 Identification of reference genes in the *Kurthia gibsonii* VITAM20 genome

For the identification of a few reference genes associated with anti-inflammatory and antioxidant activity, we implemented a customized analysis pipeline built on the BLAST+ suite and supported by automated parsing and filtering routines. Reference sequences of interest, consisting of eps, NEMO, dps, gshR3, gshR4, KatA, spa, spaB, CAT, oxyR, KatA_pseudo, perR, sodA, SodM, and trxA, were retrieved from NCBI and prepared in the FASTA format as query inputs (the query inputs are given as Supplementary Table S1). The assembled genome of *Kurthia gibsonii* VITAM20, containing nearly 2.9 million base pairs, was formatted into a searchable BLAST+ database using the “makeblastdb” utility. Query sequences were searched against this database, and alignments were retrieved in tabular format, which provided information on sequence identity, alignment length, mismatches, bit score, and statistical significance. Alignments meeting the defined quality thresholds, percentage identity ≥70%, and E-value ≤1e-5 were maintained for further analysis.

### 2.4 HRBC membrane stabilization method

Blood samples were drawn from healthy individuals and mixed with an equivalent volume of Alsever's solution (2% dextrose, 0.8% sodium citrate, 0.05% citric acid, and 0.42% sodium chloride in distilled water). For the collection of human blood, an approval from the institutional ethical committee for human studies (VIT/IECH/2025/16 IECH/15 February 2025/19) was obtained. The sample was then centrifuged for 10 min at 3,000 rpm, and concentrated red blood cells were cleaned using isotonic saline with 0.9% NaCl. A 10% suspension of packed red blood cells (HRBC) was then formulated using isotonic saline. Different volumes of the crude samples were prepared by utilizing distilled water, which involved mixing phosphate buffer (1 mL), hypotonic saline (2 mL), and HRBC solution (0.5 mL). The assay mixtures were kept constant for 30 min at 37 °C and then spun at 3,000 rpm for 20 min to collect the liquid on the top for further evaluations (Joseph et al., 2013). Diclofenac was used as the reference standard. All the experiments were carried out in triplicates (n = 3). The percentage of HRBC membrane protection was calculated using the following formula.

$$\%\;Protection=100-\left[\left(OD\;of\;test\;sample÷OD\;of\;control\right)\times 100\right].$$

The percentage of hemolysis of the HRBC membrane can be calculated using following equation.

$$\%\;Hemolysis=\left(OD\;of\;test\;sample÷OD\;of\;control\right)\times 100.$$

### 2.5 Heat-induced hemolysis

The hemolysis experiment, induced by thermal conditions, evaluated the anti-inflammatory effects of the bacterial crude sample under temperature stress. Sample concentrations of 50, 100, 200, 400, and 800 μg/mL were prepared in ethanol using isotonic phosphate buffer. Tubes were incubated for 20 min at 54 °C in a regulated water bath. After centrifuging the mixture for 5 min at 5,000 rpm, the absorbance was quantified at 560 nm. Diclofenac was used as the reference standard (Akwayamai John and Henry Bulama, 2020).

The following formula was used to calculate the percentage inhibition of hemolysis.

$$\%\;Protection=100-\left[\left(\;OD\;of\;test\;sample÷OD\;of\;control\right)\times 100\right].$$

### 2.6 Egg albumin denaturation assay

The anti-inflammatory efficiency of the crude extract sample was evaluated using the denaturation of albumin assay, following the method described by Hasan et al. (2024) with minor modifications. The reaction mixture consisted of 1 mL of phosphate buffer (pH 6.5), 200 μL of hen’s egg albumin, and various concentrations from hen’s egg albumin, and various concentrations of the crude sample. An equal volume of distilled water served as the control. The reaction mixtures then heated and incubated at 37 °C for 15 min and then later received a thermal treatment at 70 °C for a span of 5 min. The absorbance was taken after the cooling process at 660 nm. All the experiments were carried out in triplicates (n = 3). Diclofenac was used as a reference standard drug (Chaiya et al., 2022). The percentage inhibition was calculated in accordance with the following formula.

$$\%\;Inhibition=\left[1-\left(\;Absorbance\;of\;sample÷Absorbance\;of\;control\right)\right]\times 100.$$

### 2.7 Antioxidant activity assay

The antioxidant potential of the bacterial isolate was analyzed using the 2,2-diphenyl-1-picrylhydrazyl (DPPH) free radical-scavenging activity. Different volumes of the supernatant obtained from the freshly inoculated culture were mixed with DPPH (2.5 mL) solution, and the total volume was adjusted to 3 mL with the addition of distilled water. The mixture was agitated thoroughly and incubated for 30 min at 37 °C in the dark. The absorbance was later measured at 515 nm. Ascorbic acid was used as the standard reference (Pourramezan et al., 2020). The percentage of free radical scavenging activity was calculated using the following equation.

$$\%\;Inhibition=\left[\left(\;Absorbance\;of\;control-Absorbance\;of\;sample\right)÷Absorbance\;of\;control\right]\times 100.$$

### 2.8 Statistical analysis

All experiments were performed with clearly defined biological triplicates. GraphPad Prism software version 9.0.0 (GraphPad Software, San Diego, CA, United States) was used for statistical analysis for the determination of the difference among samples. The results were expressed as the mean ± standard deviation (SD). Two-way ANOVA using Sidak’s multiple comparisons test was performed.

### 2.9 Antibacterial activity

*Kurthia gibsonii* VITAM20’s antibacterial activity was analyzed against Gram-positive bacteria, especially *Staphylococcus aureus* (MTCC 3160) and *Listeria monocytogenes* (MTCC 657), along with its effects on Gram-negative bacteria including *Escherichia coli* (MTCC 443) and *Pseudomonas aeruginosa* (MTCC 2582). This experiment utilized ciprofloxacin as the standard reference. The antibacterial activity was analyzed according to the recommendations established by the National Committee for Clinical Laboratory Standards (NCCLS) utilizing the agar well diffusion procedure. The bacteria were grown in the nutrient broth for a duration of 24 h (1.5 × 10^8 CFU/mL) and swabbed onto sterile Mueller–Hinton agar plates. Following that, a sterile well punch was used to punch wells with a diameter of 6 mm, and the crude sample supernatant (100 μg/mL) was added. The agar plates were later incubated at 37 °C for a full day, using unprocessed sterile nutrient broth as the negative control for comparison (Valgas et al., 2007). The minimum inhibitory concentration (MIC) of the crude enzyme extract was determined according to the guidelines of the Clinical and Laboratory Standards Institute (CLSI), 2010 using the broth microdilution method in Mueller–Hinton broth. A bacterial inoculum (1.5 × 10^8 CFU/mL) was prepared from 24-hour-old cultures and standardized to the 0.5 McFarland standard. The crude extract was tested against *Staphylococcus aureus* and *Listeria monocytogenes*. Ciprofloxacin was used as the positive control at a concentration of 2 μg/mL, while distilled water served as the solvent control, and Mueller–Hinton broth served as the sterility control. In each well of a 96-well microplate, 100 µL of Mueller–Hinton broth, 10 µL of the standardized bacterial suspension, and 100 µL of the crude enzyme extract at concentrations ranging from 9.06 to 1000 μg/mL were added. The plates were then incubated at 37 °C for 24 h, and the optical density was measured at 600 nm. All experiments were performed in triplicate (n = 3). The MIC determination using the resazurin-based 96-well microtiter plate method is shown in the supplementary image (Al-Mijalli et al., 2023).

## 3 Results

### 3.1 Identification of VITAM20

The VITAM20 strain was identified as *Kurthia gibsonii* by whole-genome sequencing and characterization (Oxford Nanopore Technologies). The genomic DNA of the strain was successfully extracted and quantified. The NanoDrop analysis revealed a DNA concentration of 172.1 ng/μL, with A260/280 and A260/230 ratios of 1.86 and 2.29, respectively, indicating high purity of the extracted DNA. The GC content of the strain *Kurthia gibsonii* VITAM20 was 36.5%. Genome assembly was performed from scratch with Unicycler, and then scaffolding with Ragout led to the draft genome of approximately 2.97 Mb for VITAM20. BLAST-based identification results are provided in Supplementary Material to support the taxonomic placement of the strain.

### 3.2 Growth curve

**FIGURE 1:**
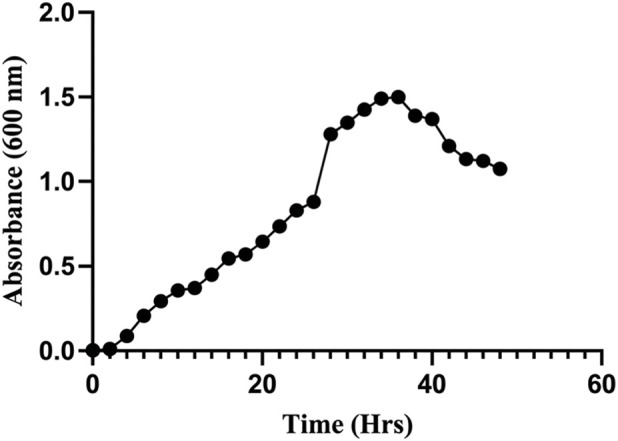
Growth curve of *Kurthia gibsonii* VITAM20.

The growth curve showed a typical microbial growth pattern with distinct phases. Initially, a lag phase was observed (0 h–4 h), during which a minimal increase in optical density indicated cellular adaptation to the new environment. This was followed by a log phase (6 h–28 h), characterized by a rapid and consistent increase in growth, which reflected active cell division and metabolic activity. The growth reached a maximum at approximately 34 h –36 h, indicating the onset of the stationary phase, where the rate of cell proliferation balanced the rate of cell death. Later, a gradual decline in growth was observed from 38 h onward, marking the death phase, during which a reduction in cell density indicated loss of viability and cell lysis. Overall, the strain exhibited a distinct growth profile with the peak biomass achieved at approximately 36 h (Figure 1).

### 3.3 Computational identification of functional gene clusters

The whole-genome characterization of the bacterial isolate revealed that it was a *Kurthia gibsonii* VITAM20 strain. To assess the presence of few reference genes responsible for the anti-inflammatory and antioxidant properties within the *Kurthia* sp. Genome, a nucleotide sequence resemblance analysis was conducted using a custom Python-based pipeline. The evaluation yielded high-confidence alignments for various target genes. The *eps* gene demonstrates 95.455% nucleotide identity, with an E-value of 0.037 and a bit score of 36.2. The *NEMO* gene also exhibits complete identity (100%) with an E-value of 0.39 and a bit score of 32.5. The *p75* gene shares 95% identity, with an E-value of 0.7 and a bit score of 32.5. The *spaA* gene shows 100% sequence identity, confirmed by a low E-value of 0.033 and a bit score of 38.1. The *spaB* gene shows the lowest percentage identity (90.625%), albeit with a highly significant E-value of 0.000686 and the highest bit score among the identified genes (43.6). The *CAT* gene exhibits 92.308% nucleotide similarity, with an E-value of 0.015 and a bit score of 38.1. The *dps* gene determines 95.238% identity (E-value 0.065, bit score 34.4), while the *gshR3* gene shares 91.304% identity (E-value 0.64, bit score 32.5). Complete sequence identity (100%) is observed for the *gshR4* gene (E-value 0.63, bit score 32.5), the *KatA* gene (E-value 0.69, bit score 32.5), and the *oxyR* gene (E-value 0.033, bit score 36.2). The *KatA* gene shows 88.462% sequence identity (E-value 0.69, bit score 32.5). The *perR* gene, although presenting a lower percentage identity of 74.041%, exhibits a highly significant E-value of 8.94 × 10^−30^ with the highest bit score among this set (126), signifying strong alignment confidence despite divergence. The *sodA* gene also displays complete identity (100%) with an E-value of 0.28 and bit score of 32.5, while the *sodM* gene shares 76.471% identity (E-value 0.022, bit score 36.2). Finally, the *trxA* gene records 77.778% identity with an E-value of 0.000886 and a bit score of 39.9.

### 3.4 Detection of secondary metabolites using antiSMASH

**FIGURE 2:**
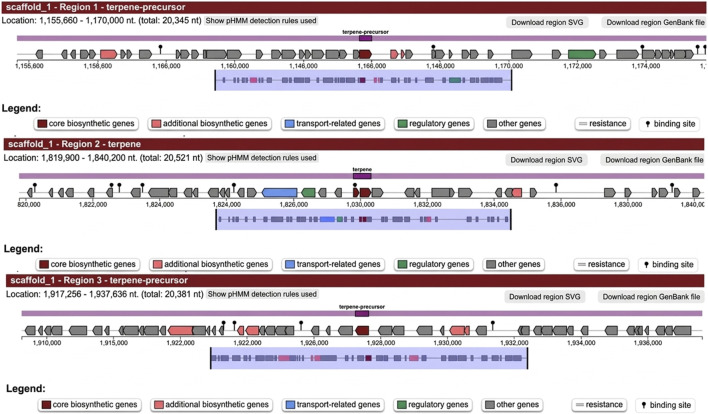
Secondary metabolite production pathways predicted by antiSMASH, including terpene-precursor and terpene.

Using the antiSMASH software designed for detecting secondary metabolites, we identified a biosynthetic pathway associated with terpenes and their precursors. In addition, we observed that there is no similarity confidence because these are categorized into unidentified clusters. These compounds contributed to the increased functionality of bacteriocins by providing additional antimicrobial metabolites. We also identified key genes that are essential to produce cyclic-lactone auto-inducers, which revealed a quorum-sensing network that influenced the activation of bacteriocin gene clusters (Figure 2).

### 3.5 Results for anti-inflammatory assays

**FIGURE 3:**
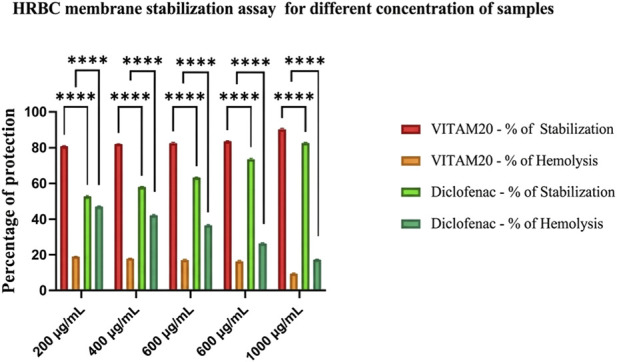
HRBC membrane stabilization assay showing the percentage protection at different concentrations of crude enzyme extract from *Kurthia gibsonii* VITAM20 and the standard drug diclofenac. All experiments were performed in triplicate (n = 3), and data are presented as the mean values.

*In vitro* studies of *Kurthia gibsonii* VITAM20 indicated that the isolate possesses remarkable anti-inflammatory properties against inflammation. The results are summarized in Figure 3. Compared to the stabilization reference outlined by diclofenac, the crude enzyme sample (1,000 μg/mL) revealed a notable stabilization of 90.78% ± 1.03%.

For the different concentrations of samples, Sidak’s multiple comparisons test (α = 0.05) showed that VITAM20 exhibited significantly higher stabilization percentages than diclofenac (group C) at all concentrations (200 μg/mL–1,000 μg/mL), with adjusted p-values <0.0001. The mean difference in stabilization ranged from 7.65% to 28.04%, indicating a stronger anti-inflammatory effect of *Kurthia gibsonii* VITAM20 compared to that of diclofenac.

**FIGURE 4:**
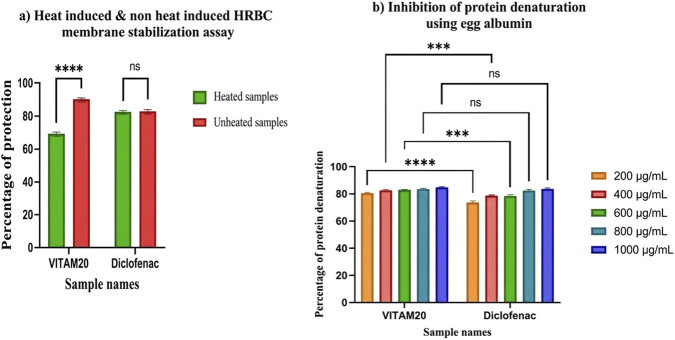
**(a)** Comparison of heat-induced and non-heat-induced hemolysis between *Kurthia gibsonii* VITAM20 crude enzyme extract and the standard drug diclofenac. **(b)** Effect of crude enzyme extract from *Kurthia gibsonii* VITAM20 on egg albumin denaturation compared with that of the standard diclofenac. All experiments were performed in triplicate (n = 3), and data are presented as the mean values.

At the tested concentrations, *Kurthia gibsonii* VITAM20 exhibited protection of 69.92% ±1.08% under heated conditions and 90.78% ± 1.03% in the absence of heat, signifying a significant degree of membrane stabilization (Figure 4A). Conversely, the reference drug diclofenac showed protection of 83.83% ± 1.23% (upon heating) and 83.01% ± 0.72% (without heating). A two-way repeated-measures ANOVA showed significant effects of treatment, heating, and their interaction. Both VITAM20 and diclofenac differed significantly in anti-inflammatory activity (p = 0.0112), while unheated samples exhibited significantly higher activity than heated samples (p = 0.0095). The significant interaction (p = 0.0122) indicates that heating affected *Kurthia gibsonii* VITAM20 more strongly than diclofenac, indicating that *Kurthia gibsonii* VITAM20 is comparatively less heat-stable.

The experiment evaluating the denaturation of egg albumin was conducted to analyze the anti-inflammatory characteristics of *Kurthia gibsonii* VITAM20 at different concentrations, with diclofenac acting as the reference. Protein denaturation was strongly increased in *Kurthia gibsonii* VITAM20 in a concentration-dependent manner, such as 80.94% ± 0.57% (200 μg/mL), 82.58% ± 0.43% (400 μg/mL), 82.7% ± 0.32% (600 μg/mL), 83.41% ± 0.32% (800 μg/mL), and 84.47% ± 0.39% (1000 μg/mL). Simultaneously, diclofenac demonstrated 74.82% ± 1.08%, 78.58% ± 0.47%, 78.35% ± 0.44%, 82.23% ± 0.44%, and 83.29% ± 0.69% increase (Figure 4B). Sidak’s multiple comparisons test found that VITAM20 showed significantly higher activity than diclofenac at 200 μg/mL, 400 μg/mL, and 600 μg/mL (*p* < 0.0001, *p* = 0.0003, and *p* = 0.0001), respectively, but no considerable difference at 800 μg/mL and 1000 μg/mL (*p* = 0.5085 and *p* = 0.2565). This demonstrates that the advantage of VITAM20 over diclofenac is prominent only at lower concentrations.

### 3.6 Antioxidant activity results

**FIGURE 5:**
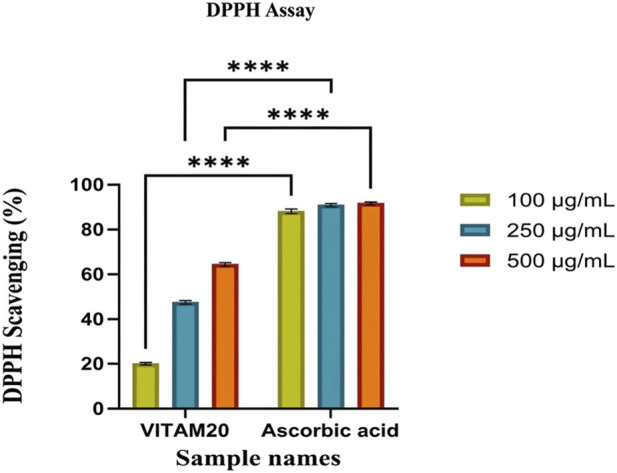
Antioxidant activity of *Kurthia gibsonii* VITAM20 extract measured by DPPH radical scavenging and expressed as the mean ± SD (n = 3).

The antioxidant effect of the crude extract of *Kurthia gibsonii* VITAM20 was systematically assessed through the DPPH radical scavenging assay. The DPPH assay demonstrated the test isolate’s ability to eliminate free radicals. All experiments were performed in triplicate, and the results are expressed as the mean ± standard deviation. The cell free supernatant sample of *Kurthia gibsonii* VITAM20 showed 64.27% ± 0.46% (500 μg/mL) of scavenging, respectively. Ascorbic acid (100 μg/mL), the positive control, showed 91.40% ± 0.46% free radical scavenging. Ascorbic acid exhibited significantly higher radical scavenging activity than *Kurthia gibsonii* VITAM20 (p = 0.0081). Increasing the concentration significantly enhanced the antioxidant activity in both the samples (p = 0.0005). A significant interaction between the sample type and concentration was also observed (p = 0.0006), indicating that the response to concentration differed between *Kurthia gibsonii* VITAM20 and ascorbic acid. Overall, ascorbic acid demonstrated superior antioxidant potential than *Kurthia gibsonii* VITAM20. The concentration required to inhibit 50% of DPPH radicals (IC_50_) was calculated by linear interpolation of the dose–response curve. The IC_50_ value of the VITAM20 extract was determined to be 286.5 μg/mL, whereas ascorbic acid exhibited a substantially lower IC_50_ value, indicating superior antioxidant potency. These findings indicate that the VITAM20 extract shows moderate free radical scavenging activity in a dose-dependent manner (Figure 5).

### 3.7 Anti-bacterial activity

The crude enzyme samples formed a 13-mm sized zone of inhibition against *Staphylococcus aureus* and *Listeria monocytogenes*, respectively (Table 1), and the zone size of the positive control was 25 mm.

**TABLE 1:** Antibacterial activity of the crude enzyme extract from *Kurthia gibsonii* VITAM20 against selected bacterial strains.

Sl. No	Concentration of the sample (µg/mL)	Test organism	Zone of inhibition (mm)
1	100	*Listeria monocytogenes*	No zone
2	100	*Staphylococcus aureus*	13 mm
3	100	*Pseudomonas aeruginosa*	No zone
4	100	*Escherichia coli*	13 mm

The antibacterial activity of the crude enzyme extract against *Listeria monocytogenes* and *Staphylococcus aureus* was evaluated using MIC. All experiments were performed in triplicate (n = 3), and the results are presented as the mean optical density (OD ± SD). A concentration-dependent inhibitory effect was observed against both test organisms. For *L. monocytogenes*, the mean OD decreased from 0.339 ± 0.001 at 9.0625 μg/mL to 0.165 ± 0.005 at 250 μg/mL. Similarly, for *S. aureus*, it exhibited a reduction from 0.375 ± 0.004 to 0.212 ± 0.001 over the same concentration range. Against *L. monocytogenes*, the percentage inhibition ranged from 61.91% at 9.0625 μg/mL to 81.46% at 250 μg/mL. Likewise, for *S. aureus*, it showed inhibition ranging from 60.36% at 9.0625 μg/mL to 77.82% at 250 μg/mL. The MIC was determined to be 250 μg/mL (0.25 mg/mL) for both pathogens. At this concentration, the crude enzyme extract inhibited the growth of *L. monocytogenes* and *S. aureus* by 81.46% and 77.82%, respectively. The MIC_50_, representing the concentration required to inhibit 50% of bacterial growth, was calculated as 72.5 μg/mL for both bacterial strains. Since 90% inhibition was not achieved within the tested concentration range, the MIC_90_ was estimated to be greater than 250 μg/mL for both organisms.

The positive control exhibited OD values of 0.240 for *L. monocytogenes* and 0.159 for *S. aureus*, whereas the sterility and solvent controls showed negligible absorbance values (<0.07), confirming the validity and reliability of the assay.

## 4 Discussion

Bacteria play an important role, including the synthesis of antimicrobial substances and various metabolites. Various studies have shown that the most effective strategy to safeguard human beings from the consequences of oxidative reactions is to preclude such reactions, which can be achieved through the utilization of antioxidants. In recent years, antioxidants that occur in nature and are extracted from biological origins have garnered notable interest as a sensible replacement for synthetic antioxidants (Faraki and Rahmani, 2020).

Considering the pharmaceutical significance and industrial demand of this enzyme, the current study was concentrated on the identification of a strain with bioactive potential. The whole-genome characterization of the bacterial isolate revealed that it was the *Kurthia gibsonii* VITAM20 strain with 2,970,044 base pairs (bp), including 6,973 predicted genes. The GC content of the strain VITAM20 (36.5%) was within the reported range for bacterial genomes (13%–75%), indicating that the strain possesses a typical genomic base composition that is consistent with previously characterized bacteria (Mesbah et al., 2011). In previous studies, *K. sibirica* NBRC 101530 possessed the largest genome size (3.4 Mb), whereas *K. gibsonii* M6 exhibited the smallest genome (2.8 Mb). The highest GC content (39.50%) was observed in *K. massiliensis* JC30, while the lowest (36.00%) was reported in *K. sibirica* NBRC 101530. In comparison, our *Kurthia gibsonii* VITAM20 exhibited a GC content of 36.5% and a genome size of 2.9 Mb, which was within the range reported for the genus (Roy et al., 2025).

The growth curve follows the lag (0 h–12 h), exponential (12 h–30 h), stationary (30 h–38 h), and decline (>38 h) stages. This agrees with normal microbial growth. Compared to other studies, the lag phase here is slightly longer, indicating slower adaptation of the cells to the medium. The exponential phase shows moderate growth rather than steep increases, indicating possible environmental or nutrient limitations. The stationary phase peak and slight fluctuation are consistent with reports of metabolic adjustments in dense cultures, while the sharp decline phase indicates nutrient depletion and accumulation of toxic byproducts, which occurs more gradually in some studies (Jones et al., 2023; Kumakura et al., 2023).

In the Planococcaceae family, *Kurthia* has an evolutionary relationship with some species such as *Lactobacillus*, *Bacillus*, *Staphylococcus*, and *Streptococcus* (Paul et al., 2022). The strain *Kurthia gibsonii* VITAM20 was isolated from a soil sample and was identified with significant bioactive properties. The analysis revealed a diverse collection of genes linked to anti-inflammatory and antioxidant functions within the bacterial strain. In addition to this, the presence of both terpene compounds and their precursors was identified by antiSMASH 7.0.

Genes associated with anti-inflammatory properties such as *eps* (95.455%), *NEMO* (100%), *spa* (100%), and *spaB* (90.625%) were identified within the genome. Especially, *luxS* and *NEMO* are important for the development of quorum sensing and the regulation of NF-κB signaling. The genes *eps* and *slpA* are substantial in the structural and interactive components of bacterial extracellular polysaccharides and surface layers and are valued for their effect on decreasing inflammatory responses. The presence of the *spa* and *spaB* genes further validates the strain’s potential in the modulation of immune responses.

*NEMO* is required for the preservation of epithelial integrity and the regulation of intestinal inflammation through NF-κB signaling and the modulation of apoptotic processes, thereby serving as a prospective therapeutic target in the modulation of inflammatory diseases (Tas et al., 2006; Nenci et al., 2007). Genes linked with antioxidant properties, such as *gshR3* (91.304%), *gshR4* (100%), *KatA* (100%), *CAT* (92.308%), *dps* (95.238%), *oxyR* (100%), *KatA_pseudo* (88.462%), *sodA* (100%), *SodM* (76.471%), *perR* (74.041% with the maximum bit score of 126), and *trxA* (77.778%), specify a robust system for the detoxification of reactive oxygen species. These genes encode enzymes such as superoxide dismutase, catalases, and thioredoxins that together function to maintain the cellular redox balance and oxidative damage. The completeness and characteristic diversity in this antioxidant gene set recommend the strain’s strong ability to withstand oxidative stress and support host health through antioxidative mechanisms. *Kurthia gibsonii* has been reported to exhibit antioxidant potential as an adaptive response to environmental stress. Certain strains, for example, *K. gibsonii* TYL-A1, showed antioxidant activity through the production of enzymes such as superoxide dismutase and catalase (Wang et al., 2024).

In an earlier study, hepatocytes and fibroblasts obtained from Tg (CAT) (+/+) mice demonstrated an increased resistance to cell death induced by hydrogen peroxide (Chen et al., 2004). The thioredoxin (Trx) system is needed for electron transfer to thiol-dependent peroxidases, supporting the effective eradication of reactive oxygen. The antioxidant function of Trx was also evidenced through its participation in DNA and protein repair mechanisms by inhibiting ribonucleotide reductase and methionine sulfoxide reductases, in addition to modulating several redox-sensitive transcription factors. Furthermore, Trx systems played a vital role in immune responses, cell death, and viral infection processes through interactions with thioredoxin-interacting proteins (Lu and Holmgren, 2014). The DNA-binding protein derived from starved cells (Dps) conferred protection against oxidative stress during growth in the exponential phase and additionally conferred resistance to UV and gamma irradiation, along with protection against harmful effects of iron and copper, thermal pressure, and acid–base shock (Nair and Finkel, 2004). Glutathione reductase (GSR), a pivotal enzyme within the glutathione antioxidant defense mechanism, catalyzes the reduction of oxidized glutathione (GSSG) to its reduced form (GSH), thus preserving the intracellular redox state to guard cellular defense against oxidative damage (Han et al., 2017). In another study, *Pseudomonas aeruginosa* OxyR was recognized as an important transcriptional regulator of the *katA* gene, capable of finding H_2_O_2_ through conserved cysteine residues and undergoing many oxidation and activation states *in vivo* (Heo et al., 2010). Together, the important enzymes capable of antioxidant resistance such as superoxide dismutase (SOD), catalase (CAT), and glutathione peroxidase (GPx), were required for the dismutation of superoxide radicals (O^2-^) and hydrogen peroxide (H_2_O_2_). SOD inhibits the synthesis of peroxynitrite and thus maintains physiologically significant levels of nitric oxide, which is necessary for vasodilation neurotransmission and inflammatory reactions (Jomova et al., 2024). The antiSMASH analysis further revealed the presence of biosynthetic gene clusters linked with terpenes and their precursors. Terpenes and their precursors emphasize the strain’s capacity to produce these important secondary metabolites. The bioactive potential of terpenes is known for their diversity, including antimicrobial, anti-inflammatory, and antioxidant potential, which could increase the genetic makeup of the strain to improve the health benefits. The presence of terpene biosynthesis pathways significantly enhances the therapeutic potential of this bacterial strain, providing biochemical importance for the observed genetic traits related to immune modulation and resistance to oxidative stress. *Kurthia gibsonii*, isolated from paneer, and the characterization of bacteriocin produced by this bacterium was well-explained in a previous study (Chauhan and Samant, 2022).

Denaturation of tissue proteins has been marked as a key component of the disease pathways tied to arthritis and inflammation. Research findings reveal that when proteins are denatured, autoantigens can develop in various types of arthritis. An increase in the absorbance of the protein hydrolysate and the reference drug, in comparison to the control group, signified the stabilization of albumin protein. Due to the structural resemblance between erythrocyte and lysosomal membranes, the HRBC assay was utilized for the *in vitro* study of anti-inflammatory potential. The stability of lysosomal membranes is significant in the regulation of inflammatory responses by inhibiting the secretion of bactericidal and proteolytic enzymes from activated neutrophils, which cause tissue damage and inflammatory processes (Wang et al., 2021; Rivera-Jiménez et al., 2022).

The *in vitro* studies on *Kurthia gibsonii* VITAM20 showed prominent antibacterial, anti-inflammatory, and antioxidant properties, thereby strengthening its potential therapeutic applications. The membrane stabilization assay showed that *Kurthia gibsonii* VITAM20 exhibited 90.78% protection under non-heated conditions and 69.92% protection under heated conditions. This confirmed that the isolate efficiently stabilizes the lysosomal membrane’s integrity by stopping the release of inflammatory mediators. The protein denaturation assay using egg albumin further confirmed the anti-inflammatory potential of *Kurthia gibsonii* VITAM20, demonstrating a concentration-dependent increase of protein denaturation across five different concentrations, with an increase of activity varying from 80.94% to 84.47%. Similar studies using egg albumin denaturation assays have validated this assay as a reliable approach for evaluating the anti-inflammatory potential of both natural and microbial agents, displaying a strong relationship with NSAIDs such as diclofenac, which served as the positive control. Previous studies have confirmed that the crude extract of *S. marcescens* VS56 exhibits significant dose-dependent HRBC membrane stabilization and shows the anti-inflammatory potential of 94.6%, which is comparable to the standard drug diclofenac (94.6%) (Nair and Subathra Devi, 2024). Although some studies have reported that HRBC protection by diclofenac differs depending on the concentration, incubation conditions, and the type of hemolysis inducer used (Parameswari et al., 2019).

The present study demonstrated that the *Kurthia gibsonii* VITAM20 extract possesses considerable antioxidant activity, as evidenced by its concentration-dependent DPPH radical scavenging ability. The increase in scavenging activity with increasing concentration indicates the presence of bioactive compounds capable of donating hydrogen atoms or electrons to neutralize free radicals. Although the antioxidant activity of the extract was lower than that of ascorbic acid, the observed IC_50_ value of 286.5 μg/mL confirms its moderate antioxidant potential.

In terms of antibacterial efficiency, the crude enzyme extracts derived from *Kurthia gibsonii* VITAM20 exhibited inhibitory activity against *Staphylococcus aureus* and *Listeria monocytogenes*, producing zones of inhibition of 13 mm. Although the zones of inhibition were moderate, these findings were significant considering previous studies that reported similar antimicrobial properties of bacterial isolates against these pathogens. The crude enzyme extract exhibited potent antibacterial activity against both *Listeria monocytogenes* and *Staphylococcus aureus*, as evidenced by the concentration-dependent reduction in bacterial growth. The identical MIC value of 250 μg/mL against both organisms indicates broad-spectrum efficacy against Gram-positive foodborne pathogens.

The higher inhibition observed against *L. monocytogenes* (81.46%) compared to *S. aureus* (77.82%) indicates a slightly greater susceptibility of *Listeria* to the bioactive compounds present in the crude extract. The MIC value obtained in the present study falls within the range considered indicative of moderate-to-strong antibacterial activity for crude natural extracts. Extracts exhibiting MIC values below 625 μg/mL are generally regarded as promising antimicrobial candidates (Zouine et al., 2024). Comparable antibacterial activities have been reported for extracellular metabolites and crude extracts from bacterial isolates. For instance, the ethyl acetate extract of *Bacillus amyloliquefaciens* VJ-1 exhibited a MIC of 25 μg/mL against *Staphylococcus aureus*, demonstrating the remarkable antimicrobial potential of Bacillus-derived bioactive compounds (Kadaikunnan et al., 2015). Likewise, cell-free supernatants from *Bacillus subtilis* KU43 effectively inhibited *Listeria monocytogenes*, with a MIC of 12.5% (v/v), further supporting the susceptibility of *Listeria* to Bacillus-derived antimicrobial metabolites (Park et al., 2023). The inability to achieve MIC_90_ within the tested concentration range indicates that concentrations above 250 μg/mL may be required for near-complete bacterial eradication. Overall, these results highlight the promising antibacterial potential of the crude enzyme extract as a natural antimicrobial agent. Further purification, characterization, and mechanistic studies are required to identify the active constituents responsible for the observed inhibitory activity and evaluate their potential applications.

The present study was limited to a preliminary evaluation using the crude supernatant, and the specific bioactive compounds responsible for the observed activity were not purified or characterized. Further validation through *in vivo* studies will be conducted in future studies. Overall, these findings indicated that the extract showed anti-inflammatory activity comparable to that of the standard drug, diclofenac, while also exhibiting moderate antibacterial activity against clinically significant pathogens. This dual functionality was consistent with previous studies on this isolate and its derived compounds, highlighting its potential as a promising source for the development of novel anti-inflammatory, antibacterial, and antioxidant agents.

## 5 Limitations of the study

The present study is preliminary in nature and has certain limitations. Genome mining was based primarily on sequence similarity and requires further validation through phylogenetic, comparative genomic, and gene expression analyses. Although significant biological activities were observed, the specific bioactive compounds responsible for these effects were not purified or structurally characterized. Furthermore, the anti-inflammatory effects were evaluated only through *in vitro* assays, and detailed mechanistic and *in vivo* studies are needed. These limitations will be addressed in future studies.

## 6 Conclusion

The presence of anti-inflammatory and antioxidant genes, combined with the terpene biosynthetic capability, underscored the *Kurthia gibsonii* VITAM20 strain’s functional potential for bioactive compound production. These genomic features indicated a strong potential for managing inflammation and oxidative stress-related conditions, requiring further experimental validation to confirm their efficacy and bioactivity *in vivo*. To the best of our knowledge, this was the first report to reveal the anti-inflammatory and antioxidant properties of *Kurthia gibsonii*.

## Data Availability

The original contributions presented in the study are publicly available. This data can be found here: BioProject: PRJNA1238542 (https://www.ncbi.nlm.nih.gov/bioproject/PRJNA1238542); BioSample: SAMN47475936 (https://www.ncbi.nlm.nih.gov/biosample/SAMN47475936); SRA: SRR35894991 (https://www.ncbi.nlm.nih.gov/sra/SRR35894991); Genome accession: CP185371.1 (https://www.ncbi.nlm.nih.gov/nuccore/CP185371.1).

## Supplementary material

SUPPLEMENTARY IMAGE 1 Determination of the minimum inhibitory concentration (MIC) of the crude enzyme extract from *Kurthia gibsonii* VITAM20 against *Staphylococcus aureus* and *Listeria monocytogenes* using the broth microdilution method.
