# Supplementary material for: Experimental analysis and genome mining for functional validation of genes associated with anti-inflammatory, antioxidant, and antibacterial activities in Kurthia gibsonii VITAM20
Source: Front Pharmacol. 2026 Jul 3;17:1799206. doi: 10.3389/fphar.2026.1799206 (PMC13375520; doi:10.3389/fphar.2026.1799206)
Supplement: Supplementary file 1 [file Table1.docx]

| **Query sequence used for detecting Anti-inflammatory and Antioxidant genes** |  |
| --- | --- |

| eps_gene | atggatcataaaaacagcgataacgaactgcgccatcgcagccatcatcatcgccatcatcgccgcaaaaaattttggcgcattttttggattgtgctgggcgtgtttctggcggtggatattattgcggtgattattgcgtggcataacattcatgtggcgaccaacaacatgtataacccgatgagcaacgaaattagcgatcgcaaagtgagcgataaactgaaagataaaaaaccgatgagcctgctgctgctgggcaccgataccggcgaatttggccgcagctataaaggccgcaccgataccattatgatgatggtgattaacccgaaaaccaacaaaaccaccgtggtgagcctgccgcgcgatatgaaagtgaacctgccggattatccggattatagcccggcgaaaattaacgcggcgtatacctatggcggcgtggatgaaaccgtgaaaaccattaaaaaatattttaacgtgccgaccgatagctatgtgatggtgaacatgggcggcctggaaaaagcgattgatcaggtgggcggcgtgaccgtgaaaagcccgctgacctttgattatgaaggctatcattttaccaaaggcgtgacctatcatatgaacggcaaaaaagcgctggcgtttagccgcatgcgctatgatgatccgaaaggcgattatggccgccaggaacgccagcgcctggtgattatggcgctgctgaaaagcagcattagctataaaaccgtggtgaaccaggcgtttctgaacagcattagcaaacagaccatgaccaacctgacctgataacatggtggcgctggcgcagaactatcgccatgcgaccgataacattaccagcgatcatgcgcagggccagggcattgggaaaacggcgtggcgtatgaaagcgtgagccaggcggaatgccagcgcattagcaacaaactgcgcgcggcgctgggcctgaaaccggaaaccctgaaaaccggcgaa |
| --- | --- |
| luxS_gene | atgggcaaagtggaaagctttgaactggatcataccaaagtgaaagcgccgtatgtgcgcctgattaccgtggaagaaggcaaaaaaggcgataaaattagcaactttgatctgcgcctggtgcagccgaacgaaaacgcgattccgaccggcggcctgcataccattgaacatctgctggcgggcctgctgcgcgatcgcattgatggctatattgattgcagcccgtttggctgccgcaccggctttcatctgctggtgtggggcaccccgagcaccaccgatgtggcgaaagcgctgaaagaagcgctggaagaaattcgcgataaaattcagtgggaagatgtgccgggcaccaccattaaaagctgcggcaactatcgcgatcatagcctgtttagcgcgaaacagtggagccgcgatattctggaaaaaggcattagcgatgatccgtttgaacgcaacgtggtggaa |
| NEMO_gene | atgaacattaacacccagtatctggtgaccgatccggaacgcctgaaagtgattggcccgaactggatgaacccgaccgaaattacctttcataacacctataacgatgcgagcgcgagcgcggaagtgcgcaacgtgcgcaacaacagcaccggcaccagctttcataccgcggtggatgattttgaagtgcagcaggtggtgccgtttgatcgcaacgcgtggcatgcgggcgatggcacctatggcgcgggcaaccgcaacagcattggcgtggaaatttgctatagcatgagcggcggcgaacgctatcgcaaagcggaactgaacgcgattgaacatattagcgatctgatggtgcgctttggcattccgattagcaaagtgaaaacccatcaggaacgcaacggcaaatattgcccgcatcgcatgctggatgaaggccgcgtgggctggtttaaagcggaatgcgaacgccgcgcgaacgaaaaacgcaacggcggcggcggcaccccgaccccgccgccggaaccgaaaccggaaccgaccccgaaaccgccgagcggcgattatgatagcagctggtttaccaaagaaaccggcacctttgtgaccaacaccaccattaaactgcgcaccgcgccgtttaccagcgcgggcgtgattgcgaccctgccggcgggcagcaccgtgaactataacggctttggcattgaatatgatggctatgtgtggattcgccagccgcgcagcaacggctatggctatctggcgaccggcgaaagcaaaggcggcaaacgcgtgaactattggggcacctttaaa |
| p75_gene | atggtggatgcgaaaaaagtgctgagcgtgaccgcgggctttgtgggcgcggcgggcctggcgaccctggcgaccggcgcgaacaccgtgagcgcgagcaccggcaccgtgaactataaaagcggcgcgaccaccgtgtggaacagcccgagctggcatcaggtgaaacgctatgtgacctttggcgaaaagtgcaggtgctgggccagaaagtggatcgcaacggcgcgacctggtataaagtgggcgataaccagtggattccgggcatttatctgaactttgatggcaaaaccgtgaccgtgcaggcgccggaacagaccgcgagccaggcgccggtgagccaggcgccggcgagccaggcgccggcgagccaggcgccggcgagccaggcggcggcgcagccggatacccagaccgcgaacattcagctgtatgtgaaaaacattggcagcgcggtgaccgtgtgggcgaccccggcgtatagccaggcgaccggccagtatctggaaggcaaccagaccctgaccgcggtggcgcagctgcaggcgaacggcgaaacctggtatcgcctggcgaacggcggctatgtgccggaacgctttgcgagcaccaccccggcgccggcgccgcagagcagcgcggcgaccagcgtggcggcgccgaccgtgccggtgagcgatgcgaccgcgagcaacgcggcggcgagcgatgcggcggcgagcagcgcggcggcgagcagcgcggcggcgagcctggcggtggcgagcgaagcggcggcgagcgtggcgaacgcgccgagcgcggcggcgagcagcgcggcggtggcgagcgcggcggcggaaagcagcaccgcggcggaaagcagcgcggcggcgagcaaagcggcggcggatagcagcgcggcggcggtgcagaccaccaccccggaaagcagcgcggcgccggcgaccacccaggtggatgcgacccaggaacagcagcagcaggcggaaccgagcattaccgtgaacgcggaagaaaccaccaacaacgcgaccccgaccccggcgccgaccccgaccccggcgccgaccccggcgccggcgccggtgaccccgagccgccaggcgaaaattcaggcggtgattagcattgcggaacagcagattggcaaaccgtatgtgtggggcggcaaaggcccgaacagctttgattgcagcggcctgatgtattatgcgtttctgaacggcgcgggcgtgaacattggcggctggaccgtgccgcaggaaagcagcggccagcaggtgagccgcagcgcgctgcagccgggcgatctgctgttttggggcggccatggcagcagctatcatgtggcgctgtatattggcggcggcaccatgattcaggcgccgcagccgggcgaaaacgtgaaatataccgcgctggcgtattttatgccggattttgcggtgcgcccgagcctg |
| slpA_gene | atgaaaaaaaacctgcgcattgtgagcgcggcggcggcggcgctgctggcggtggcgccggtggcggcgagcgcggtgagcaccgtgagcgcggcgaccaccattaacgcgagcagcagcgcgattaacaccaacaccaacgcgaaatatgatgtggatgtgaccccgagcgtgagcgcggtggcggcgaacaccgcgaacaacaccccggcgattgcgggcaacctgaccggcaccattagcgcgagctataacggcaaaacctataccgcgaacctgaaagcggataccgaaaacgcgaccattaccgcggcgggcagcaccaccgcggtgaaaccggcggaactggcggcgggcgtggcgtataccgtgaccgtgaacgatgtgagctttaactttggcagcgaaaacgcgggcaaaaccgtgaccctgggcagcgcgaacagcaacgtgaaatttaccggcaccaacagcgataaccagaccgaaaccaacgtgagcaccctgaaagtgaaactggatcagaacggcgtggcgagcctgaccaacgtgagcattgcgaacgtgtatgcgattaacaccaccgataacagcaacgtgaacttttatgatgtgaccagcggcgcgaccgtgaccaacggcgcggtgagcgtgaacgcggataaccagggccaggtgaacgtggcgaacgtggtggcggcgattaacagcaaatattttgcggcgcagtatgcggataaaaaactgaacacccgcaccgcgaacaccgaagatgcgattaaagcggcgctgaaagatcagaaaattgatgtgaacagcgtgggctattttaaagcgccgcatacctttaccgtgaacgtgaaagcgaccagcaacaccaacggcaaaagcgcgaccctgccggtggtggtgaccgtgccgaacgtggcggaaccgaccgtggcgagcgtgagcaaacgcattatgcataacgcgtattattatgataaagatgcgaaacgcgtgggcaccgatagcgtgaaacgctataacagcgtgagcgtgctgccgaacaccaccaccattaacggcaaaacctattatcaggtggtggaaaacggcaaagcggtggataaatatattaacgcggcgaacattgatggcaccaaacgcaccctgaaacataacgcgtatgtgtatgcgagcagcaaaaaacgcgcgaacaaagtggtgctgaaaaaaggcgaagtggtgaccacctatggcgcgagctatacctttaaaaacggccagaaatattataaaattggcgataacaccgataaaacctatgtgaaagtggcgaactttcgc |
| spaA_gene | atgctgtttaccattctgtttcagtttgtgggcagctttatggaactgggcagcgtgtttgcggtggcgaacgaagaagcggtggtgtttgaaaacgaagaatatggcaaagcgaccctgacctatacccagccgaaccagacccagtttgattggcagctgaactttgaacagaaagcgagcagcgaaaaacgccaggtgggctttattctgagcgaatatgcgagcgataaactggaactgccggaacaggtgaccaccgcggaaaacctggaatttaccaaaaccgatctggaaaacggcgaaaccctgtatctggcgaaagaaggcaccaacgaaaacaaaaccaccaaaattacctttagcaccaccgtgaaacagcagctggaacagtatgcgtttaaaattcagggcgtgattgtgaacgaagataccgcgcgcaacggcgaaaaaagctggaccaaagatgatctgaccaccgcgaacctgctgaaagatgaaaaaagccagaccgtgaacgtgcagaacgatattctgattcagcagaacgcggcgcgcgaaagcagcgaaaaagcggcggcgagcagcagcagcagcgcggcgagcgaaagcagcgaagcggatgatgatgcggatagcgataccgcgagcaccgaagaaaaagcggaaagcagcagcgaaaccgaagaagatgcgaccaccgaagcggaagataccaccagcctgggcgcgcagagcgcggcgcgcgcgaccaaaattagcgcgattgcggcgaccaacgcgggcgatctgattattagcgataaaatttttaacaacgcggcggatatgaaagtggaagtgagcgaaaccaaaattggcgcgaccgtgacccgcgtggatccggatgatgaaaacgcgggcaacagcaactataacagctatacccagattgaaaaagatgtgtattttaactatctgaaaggcagcgaagataccaaaggcagcggcaaagtgaacattaaatttgatagcgaaagcgaagatgatattgcggaactgaaaaaagcgaccgtggaagtgacctatccgaaagtgggctatattcgcaacagcaacaacaaactggtggaaattggcgcgaaagtgaaaattagcaacattattcgcagcccgctgaccggcagcgattgggatggcaaaggctatccgtataaatatccgatgattgatctgagcaccaacctgtttagcggcaccgtgctggatggcattagcagcagcgattggaactttaccttttttaaagtggatgatggcaccgatgtggattttagcaaactgagcaacccgtatatgacctttggcagcctgaacggccattataccgaaaacgcgagcaaaccggataccccgggcgtgtataacggcgaatttaaaggcgaatttgtgaaaagcatgagcggcgtgcagggcagcgtgagcggcagcgatagcctgattgtgaacgcgaaagaactgaaagtgtttaacagcgcgagcaacctggtgtttgaaaacgcgtataccggcaccgcgagcctgagcgaagataaagataaactgggccatccggtgtttaacaccagcgcggtgacctttggcctgagcggcaccaccaacagctttcagattggcaccggcggcggcagcagcaaccatgcgtggtttacctttgcgagcagcgcgattaccccgacccagcagaccgcgccggcgaaaaccgtgcagccgctgacccagtatgatagcgatggcaacgcgagcagcaacgaaagcggctttgatcagcgcttttataacgatctggatcgctataacgcggaaaccggcgaagaatgggcgctgctggaacagtatcgcgtggaaggccataacgcggaaaacgaaaaagaactggtggcgggcgtgccgaaaaaaagcgatcgctatgtgaccgtgggcagcgaatattattattttattaaccagccgaccattaactatgtgagcgaaggcctggtgagcccggataaagtgaccctgaccgataccctgccgattggcgtgagcctgagcgaagaaaccattaaaaacagctttaccctgtataacctggatggcagcgaactggatctgagcgatgatcagattgatattaccgcggcgaccgaaaccaccggccagaaaattatttttaccctgaccgaacagcagattaaagatattaacgaactgagcaaagaagaaaaacattatggcgatgattttagcctgcgcattaaagtgaaagtggcggaaaacgtgagcgcgggcgaaattatggaaaacgtggcgagcagcaactttagctataccaccaccgaaaaagatgaaaacggcattgaagaagaagtgattgtggattttgatgcggatagcaacccggtgcataccgtgaccaaagaagaaattaaaaccctgcagctgaaactgaaaaaaattgcgagcgatacccaggcgggcctggcggaagcgacctttaccgtgaccaaaaaagatgatagcaccgtgattagcaccgcgaccagcgataccaacggcctgctgacctttgatgaaccgctgaccgtgggcgaatataccctgaaagaaaccgtggcgccgagcggctatcagaaagtggcggatattgattttaccgtgaaagaaaacaccgatggcaaactggtggcggtggtgaaagtgggcccgaccgaaaccgaaattaacgaaaactatcagattgtggatcataaaattccgatgaacctgaaaattaaaaaaattgatgcgagcgataacaaagaactgagcggcgcggaatttaccctgaaaaaaggcgataacaccattaccggcaccaccagctggagcgatctgcagccgggcacctataccctgaccgaaaccaaagcgccgagcggctatcagctgagcgatagcgtgtataccattaccattgcgaaagatggcatgaccaccgtgcagaaagatggccagccgtatgcggatgtgagcattaacaaagatggcaacccgattagcattaccctgaccattaaaaacaacatgcagccggcgctgccggtgaccggcggcatgggcattctgccgctgctgattggcggcctgattctgatggcggcgggctggtttggctatcgcaaactgcgccag |
| spaB_gene | atgaaatcactgtatacaccgacagattattatatgattagagttccgctggttcatcaagatctgaaaaatgaaaattcacaagatattgatcaactgctgcatgatctgtgcaatgattcactgtttagagaacaaattctggtttcatcaagaacactgtatgaaacaattcatacatttctgcaagcaccggataaactgaaaggcaaaaaaaaaagaaattttcaacaagcaattctgaaatatgcaacaagaagagcaacaagaacaacaccgtttggcctgttttcatcagttggcattggctcattttcagataaaaatcatctgtcatttaatcaacattcattttataaaaaagcaagagttgatctggaatggctgtatcaactgattagaaaactggaaaatgaatatacagatagactgtcatttacactgaattcagcatgctatattaaaggcgatagagcatatctgctgtattcaacagatggcaaatcagaagaagtttcagttagagcaacatcagttttttatctgattaatgaactgtgcggcgaatcagcagcatatcaagatattattagatgcctgattgataattatccgaatacaccgattaataaaattaatcaatatgttgcagatctgattgataaagaatttctgatttcaaatctgagaccgccgatgacagtttcagatcaatttcaatatctgattgatcaagcagaatcaagacatattccgaatgaactgattcaagcatgcaaagatattcaatatcaaattgatgcatataatagaattacaattggcgaaggcgaacatcaatatctgaatctgattgaaacaatgaataaactgattaaagcatcatcaccgctgcaagttgatgcaggcctggcagattcatcaattcaactggataatgaaacatcactggcaatttcagaactggcatcaatgtttacatatatggcatcaccgtcagcaaatacactggatcatctggaaaaatatcataatgtttttctggaaagatatggctatgaaagagaagttccgctgctggaaatgctgtgctcatcaacaggcattggcgcaccggcaacatatacaaatccggcaaatgaattttttgaagaaacatcatttggcgaacaattttcaccggaaatgaaacaattttttatgagaaaatattttgaatcagttagaaaaaaagcaccgattcaactggatgatgaaacatttcatagaatttgcaattcagaaattgcagatgaagaaattccgctgtcatttgaactgaatttttttgttaaactgagaaatggcagagttaaactgtatctgggcccgaatgttggctcaacaagagcaggcaaaacatttggcagattttcacatatgtcagattcaatttcagaaattattaaaacactgcataataaagaaaaagaactgacagaatgcaatacaaaagtttgcgaactgtcaattgttccgaatcaaacaagatcaggcaatgttacaagaaatgtttcatatagagaaaaagaaatgtcactgtttacaaattcagcactgcatctgaatgattcagttaaagcagaagatattctgattggcattaataaagatcataatttttatgcaagacataaaacaacaggcgaaattctgtcatttgaatcaaatcatatgtttaatccgctgctgatgacaaatgcagttagatttctgctggaaatttcaagagatggcaaaagaaaatggaatgattttccgtggttttcaatttattcagattttaaatatattccggaaattaaatataaagaaattacactgtcatgcgaacaatggctgatttataaaaatgatctgtcaatgcattcaaatgcatcactggaagaaattaaatcaccgttttttgaatttcatagaacatatgaactgccgcaaacattttatattgttaatgcagataatagactgctgattgatattgaaaatgattgcacactggatgtttttttttgggaactgaaaaaaacaaatcataatcaaccgctgcaactggttgcagttgaacatgatgcagatgcactgatggatagaaatcaaaatgattattcaggcgaaattgttgttccgctgctgagaaaacaaccggaaaaaccgctgtatctgccggttctgaatgcaattgaaggctcaggctcagatagaattaaaatgccgtttgaagattggctgtttattaaactgtattgcaaacaaacaagagaagaagaactgattgcatttgaaattgcagatttttataatcaaatttcagatcaatatccggttagacatttttttatgagatatagagatccgaaaccgcatattagactgagatttaatggcaaagcagaagttctgtattcactgtttccgcaactgctgaattggctgaaatcactgagagaaaaaggcctggtttcagaatcagttattacacaatatgaaagagaaattgaaagatatggcggcctgtcactgatggaagcagcgaacaactgttttgcgaagattcaaaagttgttgaaatgatgattagaatgcatagaatgaagatattacaatttcaaaagaaattgcaggcatggtttcagttattcaatttctggaacatttgaactgacatttgaagaacaactgacatttctggaaagaaattcactgcaaaagaatatagaacagaatttaaaaaagatagagaaatgtatattgaaatttgcaattcagatgagattgggataatctgaaaaaaacatcagatggcggcatgctgtatgaaacactgaaacaagaaaaatggcagcagcacattatgcatttctgattaaaaaagcatttgataataaagtgaagtttattcaagaattggctcaattattcatctgcattgcaatagactgtttggcaagatagagaactggaaaataaaattctgacactgtgcagacattcactgtatgcacaagatatcaaaaaatgaatggctcactggcatggaaa |

| CAT_gene | atgaccgaaaaactgaccaccgaaaccggccagccgtgggcgaacaacgaacatagccagaccgcgggcgcgccggcccggtgctgatgcaggattataacctgctggaaaaactggcgcattttgatcgcgaacgcattccggaacgcgtggtgcatgcgaaaggcgcgggcgcgaaaggcttttttgaactggaaaacgatatgggcgaatataccaaagcggatctgtttaacggcgtgggcaaaaaaaccccggtgattctgcgctttagccaggtggcgggcgaaaaaggctatccggataccgtgcgcgatgtgcgcggctttgcgctgaaattttatacccagcagggcaactatgatctggtgggcaacaacaccccggtgttttttgtgaacgatccgctgaaatttccggattttattcatagccagaaacgcgatccgaaaaccaaccgccgcacccagaacatgcagtgggatttttgggcgcatagcccggaaagcctgcatcaggtgacctatctgatgggcgatcgcggcctgccggcgagctatcgcaccatgaacggctatggcagccatacctttaaatgggtgaaccagaacggcgaacagttttgggtgaaatatcattttattagcgatcagggcgtgaaaaacatgaccgcgcaggcggcggaaaaagcgatgagccaggatctggattatctgcaggatgatctgtatgatgcgattcaggaaaaaaactatccgagctggaccatgtatgtgcaattctgccgtatgaagaaggcctgaactataaatgggatatttttgatgtgaccaaagtgattagccataaagattatccgctgattaaagtgggcaaactgaccctgaacgaaaacccgaccaacaactttaccgatattgaagaagcggcgatgagcccggcgaactttgtgccgggcattgaaccgagcccggataaactgctgcagggccgcctgtttagctataaagatgcgcacgctatcgcctgggcgcgaactttgaagatctgccggtgaacaaaccggtggtgccggtgcataactatgaacgcgatggctatatgaaaattaacaaccagggcgcggaagtgaactatgaaccgaacgcgctgcatggcccggaagaagtgccggatgcggcgctgagcccggatagcgtgcgcggcgaaacccgcgcgcagccgtatcgctatcaggtggattataccacccaggcgggcgatctgtatcgcctgatgagcgaaccggaacaggaacgcctgattaacaccattaaagatgcgctgggccaggtgaccctgccgggcgtgaaagaactggaaattaaacagttttatgaagcggataaaaactatggcacccgcgtggcgaacgcgctgggcatgaacattgcggatattattagcgat |
| --- | --- |
| dps_gene | atgagcaccgcgaaactggtgaaaagcaaagcgaccaacctgctgtatacccgcaacgatgtgagcgatagcgaaaaaaaagcgaccgtggaactgctgaaccgccaggtgattcagtttattgatctgagcctgattaccaaacaggcgcattggaacatgcgcggcgcgaactttatgcggtgcatgaaatgctggatggctttcgcaccgcgctgattgatcatctggataccatggcggaacgcgcggtgcagctgggcggcgtggcgctgggcaccacccaggtgattaacagcaaaaccccgctgaaaagctatccgctggatattcataacgtgcaggatcatctgaaagaactggcggatcgctatgcgattgtggcgaacgatgtgcgcaaagcgattggcgaagcgaaagatgatgataccgcggatattctgaccgcggcgagccgcgatctggataaatttctgtggtttattgaaagcaacattgaa |
| gshR3_gene | atggcggattatgataccatttttattggcagcggccatgcgacctggcatgcggcggtggcgctggcgcatgcgcagcataaagtggcgattattgaagaagataccattgcgggcacctgcaccaactttggctgcgatgcgaaaattctgctggatggcccgtttgaactgaccgaacagctgaaacagtatcagggcattggcgtgaacaccaccccgaccattgattggagccagctgatggcgtataaacagcaggtgattcagccgctgagcgtgcagatgaccgcggtgtttaaacagctgggcattaccattattaccggccatggcgaactgaccgatacccataccgtgcaggtggcggatagcacctataccgcggataccattgtgattggcaccggccagcgcccggcgaaactggcgattccgggcgcggatctgatgcatgatagccgcgattttctggatctgccgaccatgccgaaacgcctgaccctgattggcgcgggcattattagcctggaatttgcgaacatggcggtgctgctgggcagcgaagtgcatattattgaatttgcggatcgcgcgctgccggcgttttatagcgaacatgtgaaaaaaatgattacccatctgcaggcggcgggcgtgcattttcattttggcgaagcgctgagccaggtgaccaaaaccgcgaccggcctgatggcgaccaccgcgagcggcctgaaaattgaaagcgatgatattattgcggcgaccggccgcattccgaacattgaacatctgggcctgaccaaagtgggcattaaaaccgatcgccatggcattattgtggatgatcatctgcgcaccagcattccgaacatttatgcgagcggcgatgtgattagcaaaaccctgccgaaactgaccccgaccgcgatttttgaaagcactatattgcgggccagctgctgggcagcaccgcggcgattgattatccggtgattccggcggtggtgtttaccctgccgcgcattgcgcaggtgggcgtgagcgtggaagcggcgcagcatgataccgaacattttcatgtgcaggcgctgccgtatgcaaactgctggcgtttcagtatcagaacgaagtggatgcggatctgcagctggtgtttgatcaggaaaactatctggtgggcgcgagcatttatggcaacggcgcgctggatctgattaacctgctgaccatgattattgcggatcatgtgagcgcgaccaccctgagccagaaaatttttgcgtttccgagcgcgagcgtgggcattattgatatgctgaccccgctgctgcatcatgat |
| gshR4_gene | atgaccaacaaatatgattatgatgtgctgtatattggcgcgggccatgcgacctttgatggcgcggcgccgctggcgaaaaccggcgtgcgcgtgggcgtgattgaaagcggcctgattggcggcacctgcccgaaccgcggctgcaacgcgaaaattaccctggatgaaccggtgaaactgacccgcgaaaccgcgcgcctgaacgatattctgagcagcgcgccgaccattaactggaccgcgaacgtggcgcataaacaggaaattattgatccgctgccggcgggcctgaccgcgcgcctggaagatggcggcgcgaccattattcatggccatgcgacctttaaagatgcgcataccgtggtggtggatgatcagcagaccattaccgcggaaaaaattgtgattgcgaccggcctgaaaccgcatcgcctggatattccgggcaccaaactggcgcatgatagcagcgattttatgaacctgaaacgcctgccgcagagcattgtgattattggcgcgggctatattggcatggaatttgcgaccattgcgaacgcggcgggcgcgcaggtgaccgtgatgctgcatggcgatcaggcgctgcgcgatttttatcagccgtttgtggcgcaggtggtggatgatctgaccgaacgcggcgtgacctttattaaaaacgcgaacgtgcaggcgtttaccaaacaggatgatcagtttcaggtgagctatggcgatcatcagcagctgaccaccgattggattctggatgcgaccggccgcattccgaacctggatggcctgggcctggatcgcattggcgtgaaatatgatcgcatggcgtgtatgtgaacgatcatctgcagaccaacgtgccgaacatttatgcggcgggcgatgtgctggcgaacgatctgccgaaagtgaccccggcggcgtattttgaaagcaaatatctgatgcgcctgtttagcggccagaccagcgcgccgattgattatccggtgattccgagcgtggtgtttaccagcccgcgcattgcgcaggcgggcatgaaaattccggcggcggaaaaagcgggcctgaccgtagcgataacgatctggcggattattggtattatcaggtgagcaaagaaccgattgcggcgagcaaacaggtgcatgatcaggatggccatctggtgggcgtgaccgaaattagcgatcaggcggaagatgcggtgaacgcgctgctgccggcgattgaatatcactggatcgcgaacagattgatcgcctgattggcatttttccgaccattggctatgcggcg  tggcatcgcgcg |
| KatA_pseudo_gene | atggaggagaagacccgcctgaccaccgccgccggcgccccggtggtggacaaccagaacgtgcagaccgccggcccgcgcggcccgatgctgctgcaggacgtgtggttcctggagaagctggcccacttcgaccgcgaggtgatcccggagcgccgcatgcacgccaagggcagcgccgcctacggcaccttcaccgtgacccacgacatcaccccgtacacccgcgccaagatcttcagccaggtgggcaagaagaccgacatgttcctgcgcttcagcaccgtggccggcgagcgcgggccgccgacgccgagcgcgacatccgcggcttcagcatgcgcttctacaccgagcagggcaactgggacctggtgggcaacaacaccccggtgttctacctgcgcgacccgctgaagttcccggacctgaaccacgtggtgaagcgcgacccgcgcaccaacctgcgcaacgccacttcaagtgggacttcttcagccacctgccggagagcctgcaccagctgaccatcgacttcagcgaccgcggcctgccgaagagctaccgccacatccacggcttcggcagccacaccttagcttcatcaacgccaacaacgagcgcttctgggtgaagttccacttcaagacccagcagggcatcgagaacctgaccaacgccgaggcgccgaggtgatcgcccaggaccgcgagagagccagcgcgacctgtacgagagcatcgagaagggcgacttcccgcgctggaagatgtagtgcagatcatgccggagaaggaggccgccacctaccgctacaacccgttcgacctgaccaaggtgtggccgcacggcgactacccgctgatcgaggtgggcttcttcgagctgaaccgcaacccggacaactacttcgccgaggtggagcaggccgccttcaccccggccaacgtggtccgggcatcggcttcagcccggacaagatgctgcagggccgcctgttcagctacggcgacgcccaccgctaccgcctgggcgtgaaccaccaccagatcccggtgaacgccgcccgctgcccgcaccaggtgtaccaccgcgacggcggcatgcgcgtggacggcaacaacgcccaccagcgcgtgacctacgagccgaacagcttcaaccagtggcaggagcagccggacttcagcgagccgccgctgagcctggagggcgccgccgaccactggaaccaccgcgtggacgacgactactacagccagccggccgccctgttccacctgttcaccgacgagcagaagcagcgcctgttcgccaacatcgccgaggacatccgcgacgtgccggagcagatccagcgccgccagatcggcctgttcctgaaggtggacccggcctacggcaagggcgtggccgacgccctgggcctgaagctggac |
| KATA_gene | atgagcagcaacaaactgaccaccagctggggcgcgccggtgggcgataaccagaacagcatgaccgcgggcagccgcggcccgaccctgattcaggatgtgcatctgctggaaaaactggcgcattttaaccgcgaacgcgtgccggaacgcgtggtgcatgcgaaaggcgcgggcgcgcatggctattttgaagtgaccaacgatgtgaccaaatataccaaagcggcgtttctgaggaagtgggcaaacgcaccccgctgtttattcgctttagcaccgtggcgggcgaactgggcagcgcggataccgtgcgcgatccgcgcggctttgcggtgaaattttataccgaagaaggcaactatgatattgtgggcaacaacaccccggtgttttttattcgcgatgcgattaaatttccggattttattcatacccagaaacgcgatccgaaaacccatctgaaaaacccgaccgcggtgtgggatttttggagcctgagcccggaaagcctgcatcaggtgaccattctgatgagcgatcgcggcattccggcgaccctgcgccatatgcatggctttggcagccatacctttaaatggaccaacgcggaaggcgaaggcgtgtggattaaatatcattttaaaaccgaacagggcgtgaaaaacctggatgtgaacaccgcggcgaaaattgcgggcgaaaacccggattatcataccgaagatctgtttaacgcgattgaaaacggcgattatccggcgtggaaactgtatgtgcagattatgccgctggaagatgcgaacacctatcgctttgatccgtttgatgtgaccaaagtgtggagccagaaagattatccgctgattgaagtgggccgcatggtgctggatcgcaacccggaaaactattttgcggaagtggaacaggcgacctttagcccgggcaccctggtgccgggcattgatgtgagcccggataaaatgctgcagggccgcctgtttgcgtatcatgatgcgcatcgctatcgcgtgggcgcgaaccatcaggcgctgccgattaaccgcgcgcgcaacaaagtgaacaactatcagcgcgatggccagatgcgctttgatgataacggcggcggcagcgtgtattatgaaccgaacagctttggcggcccgaaagaaagcccggaagataaacaggcggcgtatccggtgcagggcattgcggatagcgtgagctatgatcattatgatcattatacccaggcgggcgatctgtatcgcctgatgagcgaagatgaacgcacccgcctggtggaaaacattgtgaacgcgatgaaaccggtggaaaaagaagaaattaaactgcgccagattgaacatttttataaagcggatccggaatatggcaaacgcgtggcggaaggcctgggcctgccgattaaaaaagatagc |
| oxyR_gene | atgaacattcgcgatctggaatatctggtggcgctggcggaacatcgccattttcgccgcgcggcggatagctgccatgtgagccagccgaccctgagcggccagattcgcaaactggaagatgaactgggcgtgatgctgctggaacgcaccagccgcaaagtgctgtttacccaggcgggcatgctgctggtggatcaggcgcgcaccgtgctgcgcgaagtgaaagtgctgaaagaaatggcgagccagcagggcgaaaccatgagcggcccgctgcatattggcctgattccgaccgtgggcccgtatctgctgccgcatattattccgatgctgcatcagacctttccgaaactggaaatgtatctgcatgaagcgcagacccatcagctgctggcgcagctggatagcggcaaactggattgcgtgattctggcgctggtgaaagaaagcgaagcgtttattgaagtgccgcttttgatgaaccgatgctgctggcgatttatgaagatcatccgtgggcgaaccgcgaatgcgtgccgatggcggatctggcgggcgaaaaactgctgatgctggaagatggccattgcctgcgcgatcaggcgatgggcttttgctttgaagcgggcgcggatgaagatacccattttcgcgcgaccagcctggaaaccctgcgcaacatggtggcggcgggcagcggcattaccctgctgccggcgctggcggtgccgccggaacgcaaacgcgatggcgtggtgtatctgccgtgcattaaaccggaaccgcgccgcaccattggcctggtgtatcgcccgggcagcccgctgcgcagccgctatgaacagctggcggaagcgattcgcgcgcgcatggatggccattttgataaagtgctgaaacaggcggtg |
| perR_gene | atggcagcacatgaactgaaagaagcactggaaacactgaaagaaacaggcgttagaatacaccgcaaagacatgcaattctggaatatctggttaattcaatggcacatccgacagcagatgatatttataaagcactggaaggcaaatttccgaatatgtcagttgcaacagtttataataatctgagagtttttagagaatcaggcctggttaaagaactgacatatggcgatgcatcatcaagatttgattttgttacatcagatcattatcatgcaatttgcgaaaattgcggcaaaattgttgattttcattatccgggcctggatgaagttgaacaactggcagcacatgttacaggctttaaagtttcacatcatagactggaaatttatggcgtttgcaagaatgctcaaaaaaagaaaatcat |
| SODA_gene | atgacctttaaactgccggcgctgccgtatgaatatgtggcgctggaaccgtatattgaagcgaaaccatgcatctgcatcatgataaacatcataaaacctatgtgaccaaactgaacgcggcgctggcgaaacatccggatctggcgagcaaaagcctgcatgatctgctgacccaattgatgatctgccggaaagcctgcagaccccgattcgcaacaacgcgggcggccatgcgaaccatagctttttttggcgcattctgaccgaaaacgcgccgctgatgccgaccggcgaactgctggaaaaaattgaagcgcgctttggcacctttaaagattttcaggcggaatttaacaacgcggcgctgggcgtgtttggcagcggctgggcgtggctggtggtggatcatgatggcgaactgcagattatgaccaccccgaaccaggatagcccgattatgagcggcaaccgcccgctgattggcctggatgtgtgggaacatgcgtattatctgaaatatcagaacaaccgcattgattatattgaaaacttttggaaaattattaactggccgctggtggcggaactgatt |
| SodM_gene | atgccgcacgccctgccgccgctgccgtacgcctacgacgccctggagccgcacatcgagccctgaccatggagatccaccacagcaagcaccaccagacctacgtgaacaacctgaacgccgccctggagggcaccccgtacgccgagcagccggtggagagcctgctgcgccagctggccggcctgccggagaagctgcgcaccccggtggtgaacaacggcggcggccacgccaaccacagcctgttctggaccgtgatgagcccgcagggcggcggccgcccggacggcgacctgggccgcgccatcgacgagcagctgggcggcttcgaggccttcaaggacgccttcaccaaggccgccctgacccgcttcggcagcggctgggcctggctgagcgtgaccccgcagggcagcctgctggtggagagcagcggcaaccaggacagcccgctgatgaacggcaacaccccgatcctgggcctggacgtgtgggagcacgcctactacctgaagtaccagaaccgccgcccggagtacatcggcgccttctacaacgtgatcgactggcgcgaggtggcccgccgctacgcccaggccctggcc |
| trxA_gene | atgagcgataaaattattcatctgaccgatgatagctttgataccgatgtgctgaaagcggatggcgcgattctggtggatttttgggcggaatggtgcggcccgtgcaaaatgattgcgccgattctggatgaaattgcggatgaatatcagggcaaactgaccgtggcgaaactgaacattgatcagaacccgggcaccgcgccgaaatatggcattcgcggcattccgaccctgctgctgtttaaaaacggcgaagtggcggcgaccaaagtgggcgcgctgagcaaaggccagctgaaagaatttctggatgcgaacctggcg |
