# Supplementary figures and images for: Experimental analysis and genome mining for functional validation of genes associated with anti-inflammatory, antioxidant, and antibacterial activities in Kurthia gibsonii VITAM20
Source: Front Pharmacol. 2026 Jul 3;17:1799206. doi: 10.3389/fphar.2026.1799206 (PMC13375520; doi:10.3389/fphar.2026.1799206)

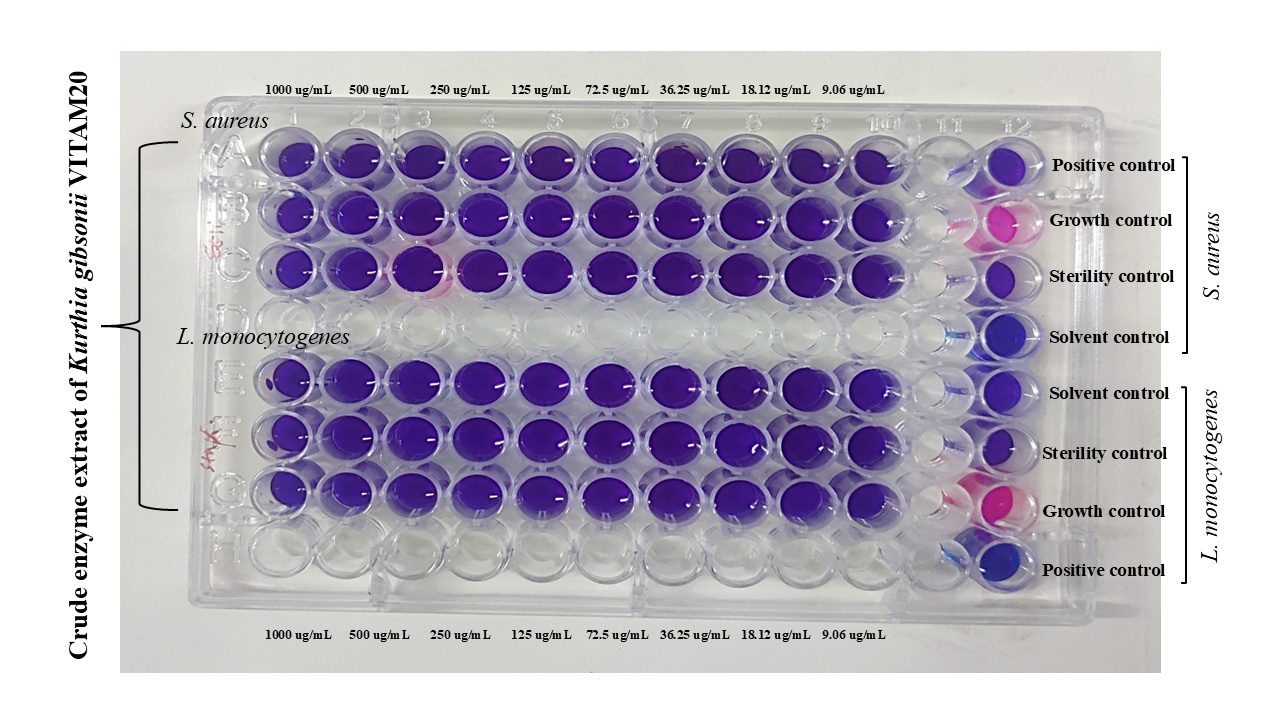

Supplement: Supplementary file 2 [file Image1.tif]
