# Supplementary material for: Experimental analysis and genome mining for functional validation of genes associated with anti-inflammatory, antioxidant, and antibacterial activities in Kurthia gibsonii VITAM20
Source: Front Pharmacol. 2026 Jul 3;17:1799206. doi: 10.3389/fphar.2026.1799206 (PMC13375520; doi:10.3389/fphar.2026.1799206)
Supplement: Supplementary file 3 [file DataSheet1.pdf]

The site is secure.
The **https://** ensures that you are connecting to the official website and that any information you provide is encrypted and transmitted securely.
Access keys NCBI Homepage MyNCBI Homepage Main Content Main Navigation

BLAST ® » **blastn suite** » **results for RID-VMB8UCZ8016**

**Job Title**	Nucleotide Sequence ...
**RID**	VMB8UCZ8016 Search expires on 03-19 12:42 pm
**Program**	BLASTN
**Database**	core_nt
**Query ID**	lcl|Query_3447041
**Description**	None ...
**Molecule type**	dna
**Query Length**	719

Descriptions

Description	Scientific Name	Max Score	Total Score	Query Cover	E value	Per. Ident	Acc. Len	Accession
Kurthia gibsonii strain KG-01 16S ribosomal RNA gene, partial sequence	Kurthia gibsonii	1251	1251	100%	0.0	98.20%	1433	PP389410.1
Kurthia gibsonii strain 1684 16S ribosomal RNA gene, partial sequence	Kurthia gibsonii	1251	1251	100%	0.0	98.20%	1455	MW405846.1
Kurthia gibsonii strain 1717 16S ribosomal RNA gene, partial sequence	Kurthia gibsonii	1251	1251	100%	0.0	98.20%	1418	MW405851.1
Kurthia gibsonii strain TB64A 16S ribosomal RNA gene, partial sequence	Kurthia gibsonii	1251	1251	100%	0.0	98.20%	1221	PV686965.1
Kurthia gibsonii strain UN-06 16S ribosomal RNA gene, partial sequence	Kurthia gibsonii	1251	1251	100%	0.0	98.20%	1387	OQ799123.1
Kurthia gibsonii strain T1G24 16S ribosomal RNA gene, partial sequence	Kurthia gibsonii	1251	1251	100%	0.0	98.20%	1469	OQ472472.1
Kurthia gibsonii strain TB71 16S ribosomal RNA gene, partial sequence	Kurthia gibsonii	1251	1251	100%	0.0	98.20%	1206	PV686972.1
Kurthia gibsonii strain IAE225 16S ribosomal RNA gene, partial sequence	Kurthia gibsonii	1251	1251	100%	0.0	98.20%	1403	MK414929.1
Kurthia gibsonii strain NCB2 16S ribosomal RNA gene, partial sequence	Kurthia gibsonii	1251	1251	100%	0.0	98.20%	758	MK813166.1
Kurthia gibsonii strain 1716 16S ribosomal RNA gene, partial sequence	Kurthia gibsonii	1251	1251	100%	0.0	98.20%	1422	MW405850.1
Kurthia gibsonii strain RCB894 16S ribosomal RNA gene, partial sequence	Kurthia gibsonii	1251	1251	100%	0.0	98.20%	1436	KT261106.1
Kurthia gibsonii strain KAU-1 16S ribosomal RNA gene, partial sequence	Kurthia gibsonii	1251	1251	100%	0.0	98.20%	1473	KT828545.1
Kurthia gibsonii strain Catla M14 16S ribosomal RNA gene, partial sequence	Kurthia gibsonii	1251	1251	100%	0.0	98.20%	1293	ON491066.1
Kurthia gibsonii strain f1-fd1_D01.ab1 16S ribosomal RNA gene, partial sequence	Kurthia gibsonii	1251	1251	100%	0.0	98.20%	995	MW534484.1
Kurthia gibsonii strain NXGG_DHWF01 16S ribosomal RNA gene, partial sequence	Kurthia gibsonii	1251	1251	100%	0.0	98.20%	1479	PV636459.1
Kurthia gibsonii strain CIFRI-AKSHG30 16S ribosomal RNA gene, partial sequence	Kurthia gibsonii	1251	1251	100%	0.0	98.20%	1443	MW301114.1
Kurthia gibsonii strain AM031 16S ribosomal RNA gene, partial sequence	Kurthia gibsonii	1251	1251	100%	0.0	98.20%	1435	OP755828.1
Bacterium peng-8 16S ribosomal RNA gene, partial sequence	bacterium peng-8	1251	1251	100%	0.0	98.20%	1394	KT698076.1
Kurthia gibsonii strain RCB321 16S ribosomal RNA gene, partial sequence	Kurthia gibsonii	1251	1251	100%	0.0	98.20%	1446	KT260533.1
Kurthia gibsonii strain SAU_AFB01 16S ribosomal RNA gene, partial sequence	Kurthia gibsonii	1251	1251	100%	0.0	98.20%	1423	MN658386.1
Kurthia gibsonii strain HBUAS71435 16S ribosomal RNA gene, partial sequence	Kurthia gibsonii	1251	1251	100%	0.0	98.20%	1470	OR186313.1
Kurthia gibsonii strain Catla C15 16S ribosomal	Kurthia gibsonii	1251	1251	100%	0.0	98.20%	1183	ON491001.1

Description	Scientific Name	Max Score	Total Score	Query Cover	E value	Per. Ident	Acc. Len	Accession
RNA gene, partial sequence								
Kurthia sp. strain Isolated_CD-F4Y 16S ribosomal RNA gene, partial sequence	Kurthia sp.	1251	1251	100%	0.0	98.20%	1419	MW418170.1
Kurthia gibsonii strain 48L4 16S ribosomal RNA gene, partial sequence	Kurthia gibsonii	1251	1251	100%	0.0	98.20%	1362	MT192444.1
Kurthia gibsonii strain L10 16S ribosomal RNA gene, partial sequence	Kurthia gibsonii	1251	1251	100%	0.0	98.20%	1231	KU315404.1
Kurthia gibsonii strain RCB309 16S ribosomal RNA gene, partial sequence	Kurthia gibsonii	1251	1251	100%	0.0	98.20%	1453	KT260521.1
Kurthia gibsonii strain 1689 16S ribosomal RNA gene, partial sequence	Kurthia gibsonii	1251	1251	100%	0.0	98.20%	1464	MW405849.1
Kurthia gibsonii strain TY-06 16S ribosomal RNA gene, partial sequence	Kurthia gibsonii	1251	1251	100%	0.0	98.20%	1417	MN960342.1
Kurthia gibsonii strain HHR10 16S ribosomal RNA gene, partial sequence	Kurthia gibsonii	1251	1251	100%	0.0	98.20%	1426	PP758843.1
Kurthia gibsonii strain TRM58103 16S ribosomal RNA gene, partial sequence	Kurthia gibsonii	1251	1251	100%	0.0	98.20%	1039	PV383805.1
Kurthia sp. SCAU15 16S ribosomal RNA gene, partial sequence	Kurthia sp. SCAU15	1251	1251	100%	0.0	98.20%	1112	KP125991.1
Kurthia gibsonii strain M25 16S ribosomal RNA gene, partial sequence	Kurthia gibsonii	1251	1251	100%	0.0	98.20%	1313	OM617813.1
Kurthia gibsonii strain VCRI-NKL 16S ribosomal RNA gene, partial sequence	Kurthia gibsonii	1251	1251	100%	0.0	98.20%	1384	PX397013.1
Kurthia gibsonii strain MF 16S ribosomal RNA gene, partial sequence	Kurthia gibsonii	1251	1251	100%	0.0	98.20%	874	KY606923.1
Kurthia gibsonii strain M15_4 16S ribosomal RNA gene, partial sequence	Kurthia gibsonii	1251	1251	100%	0.0	98.20%	1065	PV653064.1
Kurthia gibsonii strain CEMTC_6271 16S ribosomal RNA gene, partial sequence	Kurthia gibsonii	1251	1251	100%	0.0	98.20%	969	PV436835.1
Kurthia gibsonii strain KG 03/24 16S ribosomal RNA gene, partial sequence	Kurthia gibsonii	1251	1251	100%	0.0	98.20%	1374	PV147750.1
Uncultured Kurthia sp. clone K-gibsonii-1 16S ribosomal RNA gene, partial sequence	uncultured Kurthia sp.	1251	1251	100%	0.0	98.20%	1547	PQ865866.1
Kurthia gibsonii strain AM029 16S ribosomal RNA gene, partial sequence	Kurthia gibsonii	1251	1251	100%	0.0	98.20%	1417	OP755827.1
Kurthia gibsonii strain HKG391 16S ribosomal RNA gene, partial sequence	Kurthia gibsonii	1251	1251	100%	0.0	98.20%	1066	MT947182.1
Kurthia gibsonii strain KH2 16S ribosomal RNA gene, partial sequence	Kurthia gibsonii	1251	1251	100%	0.0	98.20%	1422	PP178682.1
Bacterium peng-2 16S ribosomal RNA gene, partial sequence	bacterium peng-2	1251	1251	100%	0.0	98.20%	1398	KT698070.1
Kurthia gibsonii strain gol1 16S ribosomal RNA gene, partial sequence	Kurthia gibsonii	1251	1251	100%	0.0	98.20%	1429	MK426815.1
Kurthia gibsonii strain Koac2 16S ribosomal	Kurthia gibsonii	1251	1251	100%	0.0	98.20%	1019	PQ236989.1

Description	Scientific Name	Max Score	Total Score	Query Cover	E value	Per. Ident	Acc. Len	Accession
RNA gene, partial sequence								
Kurthia gibsonii strain 1536 16S ribosomal RNA gene, partial sequence	Kurthia gibsonii	1251	1251	100%	0.0	98.20%	1443	MW405841.1
Kurthia sp. strain BG15 16S ribosomal RNA gene, partial sequence	Kurthia sp.	1251	1251	100%	0.0	98.20%	1132	OR975499.1
Kurthia gibsonii strain AM007 16S ribosomal RNA gene, partial sequence	Kurthia gibsonii	1251	1251	100%	0.0	98.20%	1444	OP755818.1
Kurthia gibsonii strain GI22 16S ribosomal RNA gene, partial sequence	Kurthia gibsonii	1251	1251	100%	0.0	98.20%	1435	PQ524612.1
Kurthia sp. B2 16S ribosomal RNA gene, partial sequence	Kurthia sp. B2	1251	1251	100%	0.0	98.20%	1348	KM391940.1
Kurthia gibsonii strain TB43 16S ribosomal RNA gene, partial sequence	Kurthia gibsonii	1251	1251	100%	0.0	98.20%	1212	PV686944.1
Kurthia gibsonii strain ZZ12 16S ribosomal RNA gene, partial sequence	Kurthia gibsonii	1251	1251	100%	0.0	98.20%	1447	KU234673.1
Kurthia gibsonii strain Molly7 16S ribosomal RNA gene, partial sequence	Kurthia gibsonii	1251	1251	100%	0.0	98.20%	1429	OL454687.1
Kurthia gibsonii strain TB65 16S ribosomal RNA gene, partial sequence	Kurthia gibsonii	1251	1251	100%	0.0	98.20%	1203	PV686966.1
Kurthia gibsonii strain PHS2 16S ribosomal RNA gene, partial sequence	Kurthia gibsonii	1251	1251	100%	0.0	98.20%	1435	ON858499.1
Kurthia gibsonii strain KH2 16S ribosomal RNA gene, partial sequence	Kurthia gibsonii	1251	1251	100%	0.0	98.20%	1480	MN453416.1
Kurthia gibsonii strain beavul.ww1 16S ribosomal RNA gene, partial sequence	Kurthia gibsonii	1251	1251	100%	0.0	98.20%	1447	OK138708.1
Uncultured bacterium clone JSP_7 16S ribosomal RNA gene, and 16S-23S ribosomal RNA intergenic spacer gene, region	uncultured bacterium	1251	1251	100%	0.0	98.20%	1519	MH444929.1
Kurthia sp. JB110_B12_VA_12EMRSA 16S ribosomal RNA gene, partial sequence	Kurthia sp. JB110_B12_VA_12EMRSA	1251	1251	100%	0.0	98.20%	1028	KU644531.1
Kurthia gibsonii strain HQB1427 16S ribosomal RNA gene, partial sequence	Kurthia gibsonii	1251	1251	100%	0.0	98.20%	928	MH044656.1
Kurthia sp. strain EB2016-75 16S ribosomal RNA gene, partial sequence	Kurthia sp.	1251	1251	100%	0.0	98.20%	1361	MN367125.1
Kurthia sp. strain NwMCC01910045 16S ribosomal RNA gene, partial sequence	Kurthia sp.	1251	1251	100%	0.0	98.20%	1394	MZ049633.1
Kurthia gibsonii strain MM10_3 16S ribosomal RNA gene, partial sequence	Kurthia gibsonii	1251	1251	100%	0.0	98.20%	1052	PV653053.1
Kurthia gibsonii strain 1682 16S ribosomal RNA gene, partial sequence	Kurthia gibsonii	1251	1251	100%	0.0	98.20%	1471	MW405844.1
Kurthia gibsonii strain PHN5 16S ribosomal RNA gene, partial sequence	Kurthia gibsonii	1251	1251	100%	0.0	98.20%	1445	ON858497.1
Kurthia gibsonii strain NOORBSRD2 16S ribosomal RNA gene, partial sequence	Kurthia gibsonii	1251	1251	100%	0.0	98.20%	1445	PP911484.1
Kurthia gibsonii strain M6 16S ribosomal RNA gene,	Kurthia gibsonii	1251	1251	100%	0.0	98.20%	1553	PP374613.1

Description	Scientific Name	Max Score	Total Score	Query Cover	E value	Per. Ident	Acc. Len	Accession
partial sequence								
Kurthia gibsonii strain BT15M 16S ribosomal RNA gene, partial sequence	Kurthia gibsonii	1251	1251	100%	0.0	98.20%	1433	OL662824.1
Kurthia gibsonii strain Y15 16S ribosomal RNA gene, partial sequence	Kurthia gibsonii	1251	1251	100%	0.0	98.20%	1427	OQ406180.1
Kurthia gibsonii strain B1 16S ribosomal RNA gene, partial sequence	Kurthia gibsonii	1251	1251	100%	0.0	98.20%	1484	PQ849185.1
Kurthia gibsonii strain 1277 16S ribosomal RNA gene, partial sequence	Kurthia gibsonii	1251	1251	100%	0.0	98.20%	1460	MW405836.1
Kurthia gibsonii strain TYL-A1 16S ribosomal RNA gene, partial sequence	Kurthia gibsonii	1251	1251	100%	0.0	98.20%	1457	OP077323.1
Kurthia sp. strain NR1 16S ribosomal RNA gene, partial sequence	Kurthia sp.	1251	1251	100%	0.0	98.20%	1036	MW036311.1
Kurthia gibsonii strain 7C 16S ribosomal RNA gene, partial sequence	Kurthia gibsonii	1251	1251	100%	0.0	98.20%	1335	MK104486.1
Kurthia gibsonii strain CBNU_45B1 16S ribosomal RNA gene, partial sequence	Kurthia gibsonii	1251	1251	100%	0.0	98.20%	1386	PP598129.1
Kurthia gibsonii strain RMS4 16S ribosomal RNA gene, partial sequence	Kurthia gibsonii	1251	1251	100%	0.0	98.20%	1523	KX950811.1
Kurthia gibsonii strain AM009 16S ribosomal RNA gene, partial sequence	Kurthia gibsonii	1251	1251	100%	0.0	98.20%	1417	OP755819.1
Kurthia gibsonii strain Rohu T23 16S ribosomal RNA gene, partial sequence	Kurthia gibsonii	1251	1251	100%	0.0	98.20%	1148	ON491161.1
Kurthia gibsonii strain B3 16S ribosomal RNA gene, partial sequence	Kurthia gibsonii	1251	1251	100%	0.0	98.20%	1480	KM391941.1
Kurthia gibsonii strain IADCAMB12 16S ribosomal RNA gene, partial sequence	Kurthia gibsonii	1251	1251	100%	0.0	98.20%	1429	MH619509.1
Kurthia sp. strain NR11 16S ribosomal RNA gene, partial sequence	Kurthia sp.	1251	1251	100%	0.0	98.20%	1047	MW036326.1
Kurthia gibsonii strain AM013 16S ribosomal RNA gene, partial sequence	Kurthia gibsonii	1251	1251	100%	0.0	98.20%	1432	OP755821.1
Kurthia sp. JB124_B12_VA_12EMRSA 16S ribosomal RNA gene, partial sequence	Kurthia sp. JB124_B12_VA_12EMRSA	1251	1251	100%	0.0	98.20%	1021	KU644530.1
Bacterium peng-9 16S ribosomal RNA gene, partial sequence	bacterium peng-9	1251	1251	100%	0.0	98.20%	1373	KT698077.1
Kurthia gibsonii strain PHB2 16S ribosomal RNA gene, partial sequence	Kurthia gibsonii	1251	1251	100%	0.0	98.20%	1432	ON858493.1
Kurthia gibsonii strain Catla C24 16S ribosomal RNA gene, partial sequence	Kurthia gibsonii	1251	1251	100%	0.0	98.20%	1356	ON491019.1
Kurthia gibsonii strain TB62 16S ribosomal RNA gene, partial sequence	Kurthia gibsonii	1251	1251	100%	0.0	98.20%	1226	PV686963.1
Kurthia sp. 11kri321 chromosome, complete genome	Kurthia sp. 11kri321	1251	11250	100%	0.0	98.20%	2964527	CP013217.1
Kurthia gibsonii strain 1685 16S ribosomal RNA gene, partial sequence	Kurthia gibsonii	1251	1251	100%	0.0	98.20%	1472	MW405847.1

Description	Scientific Name	Max Score	Total Score	Query Cover	E value	Per. Ident	Acc. Len	Accession
Kurthia sp. strain NR19 16S ribosomal RNA gene, partial sequence	Kurthia sp.	1251	1251	100%	0.0	98.20%	880	MW036334.1
Kurthia huakuii strain FPM-CIP-1 16S ribosomal RNA gene, partial sequence	Kurthia huakuii	1251	1251	100%	0.0	98.20%	1416	KY616642.1
Kurthia gibsonii strain Goldfish T11 16S ribosomal RNA gene, partial sequence	Kurthia gibsonii	1251	1251	100%	0.0	98.20%	1393	ON491101.1
Kurthia gibsonii strain ND2 chromosome, complete genome	Kurthia gibsonii	1251	11193	100%	0.0	98.20%	3006244	CP147847.1
Kurthia gibsonii strain koi1 16S ribosomal RNA gene, partial sequence	Kurthia gibsonii	1251	1251	100%	0.0	98.20%	1429	MK426822.1
Kurthia gibsonii strain SeqA3 16S ribosomal RNA gene, partial sequence	Kurthia gibsonii	1251	1251	100%	0.0	98.20%	1404	MK294240.1
Kurthia gibsonii strain HBUAS82078 16S ribosomal RNA gene, partial sequence	Kurthia gibsonii	1251	1251	100%	0.0	98.20%	1470	PV991465.1
Kurthia gibsonii strain CEMTC_7009 16S ribosomal RNA gene, partial sequence	Kurthia gibsonii	1251	1251	100%	0.0	98.20%	967	PV436836.1
Kurthia gibsonii strain TB70 16S ribosomal RNA gene, partial sequence	Kurthia gibsonii	1251	1251	100%	0.0	98.20%	1216	PV686971.1
Kurthia gibsonii strain CIFRI.CH6 16S ribosomal RNA gene, partial sequence	Kurthia gibsonii	1251	1251	100%	0.0	98.20%	1413	PV478494.1
Kurthia gibsonii strain AM001 16S ribosomal RNA gene, partial sequence	Kurthia gibsonii	1251	1251	100%	0.0	98.20%	1415	OP755830.1
Kurthia gibsonii strain S3GA 16S ribosomal RNA gene, partial sequence	Kurthia gibsonii	1245	1245	100%	0.0	98.06%	832	OP824657.1

Graphic Summary

Distribution of the top 116 Blast Hits on 100 subject sequences

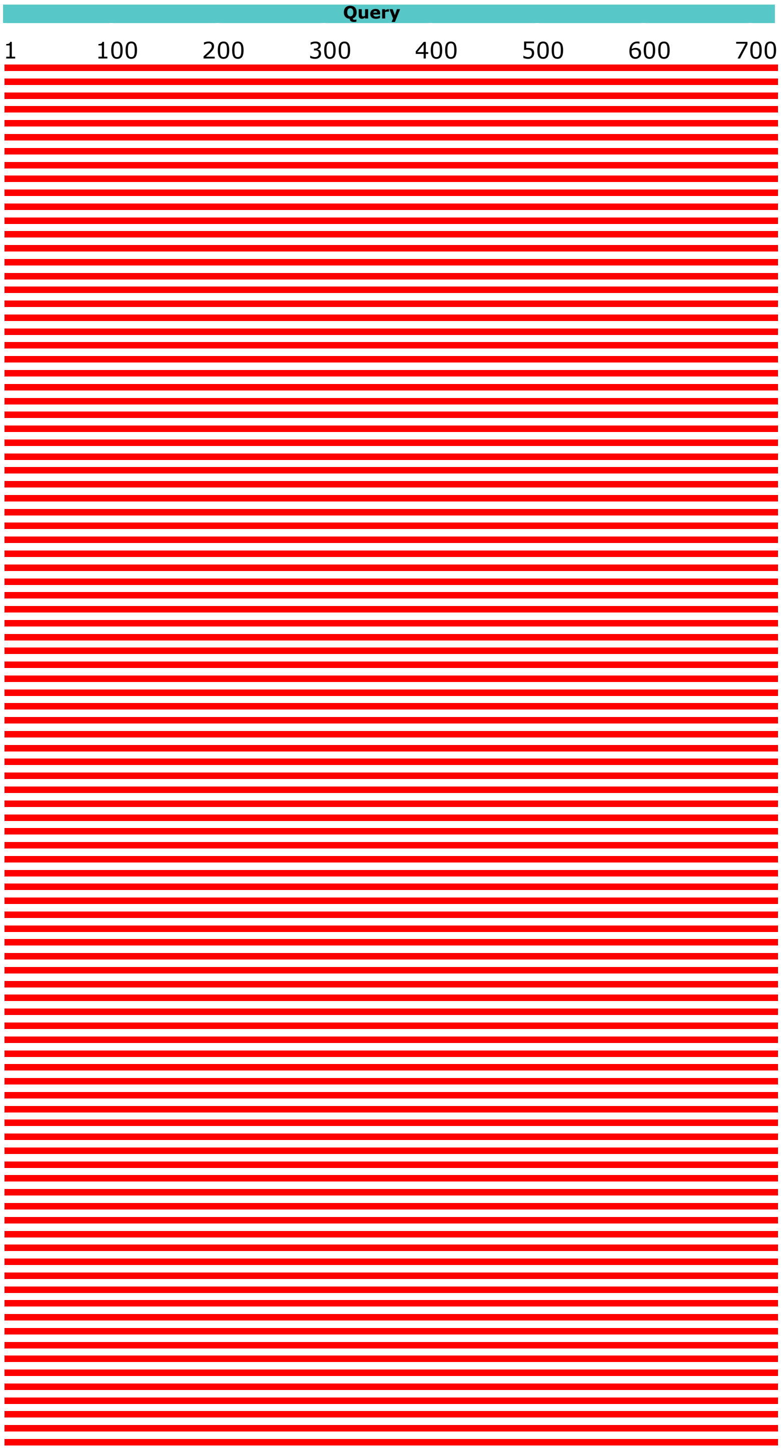

Alignments

Alignment view Pairwise ☐ CDS feature Restore defaults

Kurthia gibsonii strain KG-01 16S ribosomal RNA gene, partial sequence
Sequence ID: **PP389410.1** Length: 1433 Number of Matches: 1
Range 1: 99 to 813

Score	Expect	Identities	Gaps	Strand	Frame
1251 bits(677)	0.0()	709/722(98%)	12/722(1%)	Plus/Minus	

```
Query  3    TTAGCTGGCAGCACTAAGGGGGCGGGAAACCCCCTAACACTTAGCACTCATCGTTTACGG  62
            |||||| |||||||||| ||||| ||||||||||||||||||||||||||||||||||||
Sbjct  813  TTAGCT-GCAGCACTAA-GGGGC-GGAAACCCCCTAACACTTAGCACTCATCGTTTACGG  757

Query  63   CGTGGACTACCAGGGTATCTAATCCTGTTTGCTCCCCACGCTT-CGCGCCCTCAGTGTCA  121
            ||||||||||||||||||||||||||||||||||||||||||| |||| |||||||||||
Sbjct  756  CGTGGACTACCAGGGTATCTAATCCTGTTTGCTCCCCACGCTTTCGCG-CCTCAGTGTCA  698

Query  122  GTTACAGACCAGACAGTCGCCTTCGCCACTGGTGTTCCTCCAAATCTCTACGCATTTCAC  181
            ||||||||||||||||||||||||||||||||||||||||||||||||||||||||||||
Sbjct  697  GTTACAGACCAGACAGTCGCCTTCGCCACTGGTGTTCCTCCAAATCTCTACGCATTTCAC  638

Query  182  CGCTACACTTGGAATTCCACTATCCTCTTCTGCACTCAAGTTCCCCAGTTTCCAATGACC  241
            ||||||||||||||||||||||||||||||||||||||||||||||||||||||||||||
```

```
Sbjct  637   CGCTACACTTGGAATTCCACTATCCTCTTCTGCACTCAAGTTCCCCAGTTTCCAATGACC  578

Query  242   CTCCACGGTTGAGCCGTGGGCTTTCACATCAGACTTAAGAAACCACCTGCGCGCGCTTTA  301
             ||||||||||||||||||||||||||||||||||||||||||||||||||||||||||||
Sbjct  577   CTCCACGGTTGAGCCGTGGGCTTTCACATCAGACTTAAGAAACCACCTGCGCGCGCTTTA  518

Query  302   CGCCCAATAAATCCGGACAACGCTTGCCACCTACGTATTACCGCGGCTGCTGGCACGTAG  361
             ||||||||||||||||||||||||||||||||||||||||||||||||||||||||||||
Sbjct  517   CGCCCAATAAATCCGGACAACGCTTGCCACCTACGTATTACCGCGGCTGCTGGCACGTAG  458

Query  362   TTAGCCGTGGCTTTCTAATAAGGTACCGTCAAGGTACGTTCATTTTCCTAACGTACTTGT  421
             ||||||||||||||||||||||||||||||||||||||||||||| ||||||||||||||
Sbjct  457   TTAGCCGTGGCTTTCTAATAAGGTACCGTCAAGGTACGTTCATTT-CCTAACGTACTTGT  399

Query  422   TCTTCCCTTACAACAGAGTTTTACGATCCGAAAACCTTCATCACTCACGCGGCGTTGCTC  481
             ||||||||||||||||||||||||||||||||||||||||||||||||||||||||||||
Sbjct  398   TCTTCCCTTACAACAGAGTTTTACGATCCGAAAACCTTCATCACTCACGCGGCGTTGCTC  339

Query  482   CATCAGACTTTCGTC-ATTGTGGAAGATTCC-TACTGCTGCCTCCCGTAGGAGTTTGGGC  539
             ||||||||||||||| ||||||||||||||| ||||||||||||||||||||||||||||
Sbjct  338   CATCAGACTTTCGTCCATTGTGGAAGATTCCCTACTGCTGCCTCCCGTAGGAGTTTGGGC  279

Query  540   CGTGTCTCAGTCCCAATGTGGCCGATCACCCTCTCAGGTCGGCTATGCATCGTTGCCTTG  599
             ||||||||||||||||||||||||||||||||||||||||||||||||||||||||||||
Sbjct  278   CGTGTCTCAGTCCCAATGTGGCCGATCACCCTCTCAGGTCGGCTATGCATCGTTGCCTTG  219

Query  600   GTAGGCCGTTACCCCCACCAACTAGCTAATGCACCGCGGGGCCCAT-CTACAGTGACGCC  658
             ||||||||||||||| |||||||||||||||||||||||| ||||| |||||||||||||
Sbjct  218   GTAGGCCGTTACCCC-ACCAACTAGCTAATGCACCGCGGG-CCCATCCTACAGTGACGCC  161

Query  659   GAAGCGCCTTTCAACTTCAAAACATGTGATTCGAAGGATTATCCGGG-TTAGCCCAGGTT  717
             ||||||||||||||||||||||||||||||||||||||||||||||  ||||||||||||
Sbjct  160   GAAGCGCCTTTCAACTTCAAAACATGTGATTCGAAGGATTATCCGGTATTAGCCCAGGTT  101

Query  718   TC  719
             ||
Sbjct  100   TC  99
```

Kurthia gibsonii strain 1684 16S ribosomal RNA gene, partial sequence

Sequence ID: **MW405846.1** Length: 1455 Number of Matches: 1

Range 1: 135 to 849

Score	Expect	Identities	Gaps	Strand	Frame
1251 bits(677)	0.0()	709/722(98%)	12/722(1%)	Plus/Minus	

```
Query  3     TTAGCTGGCAGCACTAAGGGGGCGGGAAACCCCCTAACACTTAGCACTCATCGTTTACGG  62
             |||||| |||||||||| ||||| ||||||||||||||||||||||||||||||||||||
Sbjct  849   TTAGCT-GCAGCACTAA-GGGGC-GGAAACCCCCTAACACTTAGCACTCATCGTTTACGG  793

Query  63    CGTGGACTACCAGGGTATCTAATCCTGTTTGCTCCCCACGCTT-CGCGCCCTCAGTGTCA  121
             ||||||||||||||||||||||||||||||||||||||||||| |||| |||||||||||
Sbjct  792   CGTGGACTACCAGGGTATCTAATCCTGTTTGCTCCCCACGCTTTCGCG-CCTCAGTGTCA  734

Query  122   GTTACAGACCAGACAGTCGCCTTCGCCACTGGTGTTCCTCCAAATCTCTACGCATTTCAC  181
             ||||||||||||||||||||||||||||||||||||||||||||||||||||||||||||
Sbjct  733   GTTACAGACCAGACAGTCGCCTTCGCCACTGGTGTTCCTCCAAATCTCTACGCATTTCAC  674

Query  182   CGCTACACTTGGAATTCCACTATCCTCTTCTGCACTCAAGTTCCCCAGTTTCCAATGACC  241
             ||||||||||||||||||||||||||||||||||||||||||||||||||||||||||||
Sbjct  673   CGCTACACTTGGAATTCCACTATCCTCTTCTGCACTCAAGTTCCCCAGTTTCCAATGACC  614

Query  242   CTCCACGGTTGAGCCGTGGGCTTTCACATCAGACTTAAGAAACCACCTGCGCGCGCTTTA  301
             ||||||||||||||||||||||||||||||||||||||||||||||||||||||||||||
Sbjct  613   CTCCACGGTTGAGCCGTGGGCTTTCACATCAGACTTAAGAAACCACCTGCGCGCGCTTTA  554

Query  302   CGCCCAATAAATCCGGACAACGCTTGCCACCTACGTATTACCGCGGCTGCTGGCACGTAG  361
             ||||||||||||||||||||||||||||||||||||||||||||||||||||||||||||
Sbjct  553   CGCCCAATAAATCCGGACAACGCTTGCCACCTACGTATTACCGCGGCTGCTGGCACGTAG  494

Query  362   TTAGCCGTGGCTTTCTAATAAGGTACCGTCAAGGTACGTTCATTTTCCTAACGTACTTGT  421
             ||||||||||||||||||||||||||||||||||||||||||||| ||||||||||||||
Sbjct  493   TTAGCCGTGGCTTTCTAATAAGGTACCGTCAAGGTACGTTCATTT-CCTAACGTACTTGT  435

Query  422   TCTTCCCTTACAACAGAGTTTTACGATCCGAAAACCTTCATCACTCACGCGGCGTTGCTC  481
             ||||||||||||||||||||||||||||||||||||||||||||||||||||||||||||
Sbjct  434   TCTTCCCTTACAACAGAGTTTTACGATCCGAAAACCTTCATCACTCACGCGGCGTTGCTC  375

Query  482   CATCAGACTTTCGTC-ATTGTGGAAGATTCC-TACTGCTGCCTCCCGTAGGAGTTTGGGC  539
             ||||||||||||||| ||||||||||||||| ||||||||||||||||||||||||||||
Sbjct  374   CATCAGACTTTCGTCCATTGTGGAAGATTCCCTACTGCTGCCTCCCGTAGGAGTTTGGGC  315

Query  540   CGTGTCTCAGTCCCAATGTGGCCGATCACCCTCTCAGGTCGGCTATGCATCGTTGCCTTG  599
             ||||||||||||||||||||||||||||||||||||||||||||||||||||||||||||
Sbjct  314   CGTGTCTCAGTCCCAATGTGGCCGATCACCCTCTCAGGTCGGCTATGCATCGTTGCCTTG  255

Query  600   GTAGGCCGTTACCCCCACCAACTAGCTAATGCACCGCGGGGCCCAT-CTACAGTGACGCC  658
             ||||||||||||||| |||||||||||||||||||||||| ||||| |||||||||||||
Sbjct  254   GTAGGCCGTTACCCC-ACCAACTAGCTAATGCACCGCGGG-CCCATCCTACAGTGACGCC  197

Query  659   GAAGCGCCTTTCAACTTCAAAACATGTGATTCGAAGGATTATCCGGG-TTAGCCCAGGTT  717
             ||||||||||||||||||||||||||||||||||||||||||||||  ||||||||||||
Sbjct  196   GAAGCGCCTTTCAACTTCAAAACATGTGATTCGAAGGATTATCCGGTATTAGCCCAGGTT  137

Query  718   TC  719
             ||
Sbjct  136   TC  135
```

Kurthia gibsonii strain 1717 16S ribosomal RNA gene, partial sequence

Sequence ID: **MW405851.1** Length: 1418 Number of Matches: 1

Range 1: 135 to 849

Score	Expect	Identities	Gaps	Strand	Frame
1251 bits(677)	0.0()	709/722(98%)	12/722(1%)	Plus/Minus	

```
Query  3    TTAGCTGGCAGCACTAAGGGGGCGGGAAACCCCCTAACACTTAGCACTCATCGTTTACGG  62
            |||||| |||||||||| ||||| ||||||||||||||||||||||||||||||||||||
Sbjct  849  TTAGCT-GCAGCACTAA-GGGGC-GGAAACCCCCTAACACTTAGCACTCATCGTTTACGG  793

Query  63   CGTGGACTACCAGGGTATCTAATCCTGTTTGCTCCCCACGCTT-CGCGCCCTCAGTGTCA  121
            ||||||||||||||||||||||||||||||||||||||||||| |||| |||||||||||
Sbjct  792  CGTGGACTACCAGGGTATCTAATCCTGTTTGCTCCCCACGCTTTCGCG-CCTCAGTGTCA  734

Query  122  GTTACAGACCAGACAGTCGCCTTCGCCACTGGTGTTCCTCCAAATCTCTACGCATTTCAC  181
            ||||||||||||||||||||||||||||||||||||||||||||||||||||||||||||
Sbjct  733  GTTACAGACCAGACAGTCGCCTTCGCCACTGGTGTTCCTCCAAATCTCTACGCATTTCAC  674

Query  182  CGCTACACTTGGAATTCCACTATCCTCTTCTGCACTCAAGTTCCCCAGTTTCCAATGACC  241
            ||||||||||||||||||||||||||||||||||||||||||||||||||||||||||||
Sbjct  673  CGCTACACTTGGAATTCCACTATCCTCTTCTGCACTCAAGTTCCCCAGTTTCCAATGACC  614

Query  242  CTCCACGGTTGAGCCGTGGGCTTTCACATCAGACTTAAGAAACCACCTGCGCGCGCTTTA  301
            ||||||||||||||||||||||||||||||||||||||||||||||||||||||||||||
Sbjct  613  CTCCACGGTTGAGCCGTGGGCTTTCACATCAGACTTAAGAAACCACCTGCGCGCGCTTTA  554

Query  302  CGCCCAATAAATCCGGACAACGCTTGCCACCTACGTATTACCGCGGCTGCTGGCACGTAG  361
            ||||||||||||||||||||||||||||||||||||||||||||||||||||||||||||
Sbjct  553  CGCCCAATAAATCCGGACAACGCTTGCCACCTACGTATTACCGCGGCTGCTGGCACGTAG  494

Query  362  TTAGCCGTGGCTTTCTAATAAGGTACCGTCAAGGTACGTTCATTTTCCTAACGTACTTGT  421
            ||||||||||||||||||||||||||||||||||||||||||||| ||||||||||||||
Sbjct  493  TTAGCCGTGGCTTTCTAATAAGGTACCGTCAAGGTACGTTCATTT-CCTAACGTACTTGT  435

Query  422  TCTTCCCTTACAACAGAGTTTTACGATCCGAAAACCTTCATCACTCACGCGGCGTTGCTC  481
            ||||||||||||||||||||||||||||||||||||||||||||||||||||||||||||
Sbjct  434  TCTTCCCTTACAACAGAGTTTTACGATCCGAAAACCTTCATCACTCACGCGGCGTTGCTC  375

Query  482  CATCAGACTTTCGTC-ATTGTGGAAGATTCC-TACTGCTGCCTCCCGTAGGAGTTTGGGC  539
            ||||||||||||||| ||||||||||||||| ||||||||||||||||||||||||||||
Sbjct  374  CATCAGACTTTCGTCCATTGTGGAAGATTCCCTACTGCTGCCTCCCGTAGGAGTTTGGGC  315

Query  540  CGTGTCTCAGTCCCAATGTGGCCGATCACCCTCTCAGGTCGGCTATGCATCGTTGCCTTG  599
            ||||||||||||||||||||||||||||||||||||||||||||||||||||||||||||
Sbjct  314  CGTGTCTCAGTCCCAATGTGGCCGATCACCCTCTCAGGTCGGCTATGCATCGTTGCCTTG  255

Query  600  GTAGGCCGTTACCCCCACCAACTAGCTAATGCACCGCGGGGCCCAT-CTACAGTGACGCC  658
            ||||||||||||||| |||||||||||||||||||||||| ||||| |||||||||||||
Sbjct  254  GTAGGCCGTTACCCC-ACCAACTAGCTAATGCACCGCGGG-CCCATCCTACAGTGACGCC  197

Query  659  GAAGCGCCTTTCAACTTCAAAACATGTGATTCGAAGGATTATCCGGG-TTAGCCCAGGTT  717
            ||||||||||||||||||||||||||||||||||||||||||||||  ||||||||||||
Sbjct  196  GAAGCGCCTTTCAACTTCAAAACATGTGATTCGAAGGATTATCCGGTATTAGCCCAGGTT  137

Query  718  TC  719
            ||
Sbjct  136  TC  135
```

Kurthia gibsonii strain TB64A 16S ribosomal RNA gene, partial sequence

Sequence ID: **PV686965.1** Length: 1221 Number of Matches: 1

Range 1: 121 to 835

Score	Expect	Identities	Gaps	Strand	Frame
1251 bits(677)	0.0()	709/722(98%)	12/722(1%)	Plus/Minus	

```
Query  3    TTAGCTGGCAGCACTAAGGGGGCGGGAAACCCCCTAACACTTAGCACTCATCGTTTACGG  62
            |||||| |||||||||| ||||| ||||||||||||||||||||||||||||||||||||
Sbjct  835  TTAGCT-GCAGCACTAA-GGGGC-GGAAACCCCCTAACACTTAGCACTCATCGTTTACGG  779

Query  63   CGTGGACTACCAGGGTATCTAATCCTGTTTGCTCCCCACGCTT-CGCGCCCTCAGTGTCA  121
            ||||||||||||||||||||||||||||||||||||||||||| |||| |||||||||||
Sbjct  778  CGTGGACTACCAGGGTATCTAATCCTGTTTGCTCCCCACGCTTTCGCG-CCTCAGTGTCA  720

Query  122  GTTACAGACCAGACAGTCGCCTTCGCCACTGGTGTTCCTCCAAATCTCTACGCATTTCAC  181
            ||||||||||||||||||||||||||||||||||||||||||||||||||||||||||||
Sbjct  719  GTTACAGACCAGACAGTCGCCTTCGCCACTGGTGTTCCTCCAAATCTCTACGCATTTCAC  660

Query  182  CGCTACACTTGGAATTCCACTATCCTCTTCTGCACTCAAGTTCCCCAGTTTCCAATGACC  241
            ||||||||||||||||||||||||||||||||||||||||||||||||||||||||||||
Sbjct  659  CGCTACACTTGGAATTCCACTATCCTCTTCTGCACTCAAGTTCCCCAGTTTCCAATGACC  600

Query  242  CTCCACGGTTGAGCCGTGGGCTTTCACATCAGACTTAAGAAACCACCTGCGCGCGCTTTA  301
            ||||||||||||||||||||||||||||||||||||||||||||||||||||||||||||
Sbjct  599  CTCCACGGTTGAGCCGTGGGCTTTCACATCAGACTTAAGAAACCACCTGCGCGCGCTTTA  540

Query  302  CGCCCAATAAATCCGGACAACGCTTGCCACCTACGTATTACCGCGGCTGCTGGCACGTAG  361
            ||||||||||||||||||||||||||||||||||||||||||||||||||||||||||||
Sbjct  539  CGCCCAATAAATCCGGACAACGCTTGCCACCTACGTATTACCGCGGCTGCTGGCACGTAG  480

Query  362  TTAGCCGTGGCTTTCTAATAAGGTACCGTCAAGGTACGTTCATTTTCCTAACGTACTTGT  421
            ||||||||||||||||||||||||||||||||||||||||||||| ||||||||||||||
Sbjct  479  TTAGCCGTGGCTTTCTAATAAGGTACCGTCAAGGTACGTTCATTT-CCTAACGTACTTGT  421

Query  422  TCTTCCCTTACAACAGAGTTTTACGATCCGAAAACCTTCATCACTCACGCGGCGTTGCTC  481
            ||||||||||||||||||||||||||||||||||||||||||||||||||||||||||||
Sbjct  420  TCTTCCCTTACAACAGAGTTTTACGATCCGAAAACCTTCATCACTCACGCGGCGTTGCTC  361

Query  482  CATCAGACTTTCGTC-ATTGTGGAAGATTCC-TACTGCTGCCTCCCGTAGGAGTTTGGGC  539
            ||||||||||||||| ||||||||||||||| ||||||||||||||||||||||||||||
Sbjct  360  CATCAGACTTTCGTCCATTGTGGAAGATTCCCTACTGCTGCCTCCCGTAGGAGTTTGGGC  301

Query  540  CGTGTCTCAGTCCCAATGTGGCCGATCACCCTCTCAGGTCGGCTATGCATCGTTGCCTTG  599
            ||||||||||||||||||||||||||||||||||||||||||||||||||||||||||||
Sbjct  300  CGTGTCTCAGTCCCAATGTGGCCGATCACCCTCTCAGGTCGGCTATGCATCGTTGCCTTG  241

Query  600  GTAGGCCGTTACCCCCACCAACTAGCTAATGCACCGCGGGGCCCAT-CTACAGTGACGCC  658
```

```
            ||||||||||||||| |||||||||||||||||||||||| ||||| |||||||||||||
Sbjct  240  GTAGGCCGTTACCCC-ACCAACTAGCTAATGCACCGCGGG-CCCATCCTACAGTGACGCC  183

Query  659  GAAGCGCCTTTCAACTTCAAAACATGTGATTCGAAGGATTATCCGGG-TTAGCCCAGGTT  717
            ||||||||||||||||||||||||||||||||||||||||||||||  ||||||||||||
Sbjct  182  GAAGCGCCTTTCAACTTCAAAACATGTGATTCGAAGGATTATCCGGTATTAGCCCAGGTT  123

Query  718  TC  719
            ||
Sbjct  122  TC  121
```

Kurthia gibsonii strain UN-06 16S ribosomal RNA gene, partial sequence

Sequence ID: **OQ799123.1** Length: 1387 Number of Matches: 1

Range 1: 96 to 810

Score	Expect	Identities	Gaps	Strand	Frame
1251 bits(677)	0.0()	709/722(98%)	12/722(1%)	Plus/Minus	

```
Query  3    TTAGCTGGCAGCACTAAGGGGGCGGGAAACCCCCTAACACTTAGCACTCATCGTTTACGG  62
            |||||| |||||||||| ||||| ||||||||||||||||||||||||||||||||||||
Sbjct  810  TTAGCT-GCAGCACTAA-GGGGC-GGAAACCCCCTAACACTTAGCACTCATCGTTTACGG  754

Query  63   CGTGGACTACCAGGGTATCTAATCCTGTTTGCTCCCCACGCTT-CGCGCCCTCAGTGTCA  121
            ||||||||||||||||||||||||||||||||||||||||||| |||| |||||||||||
Sbjct  753  CGTGGACTACCAGGGTATCTAATCCTGTTTGCTCCCCACGCTTTCGCG-CCTCAGTGTCA  695

Query  122  GTTACAGACCAGACAGTCGCCTTCGCCACTGGTGTTCCTCCAAATCTCTACGCATTTCAC  181
            ||||||||||||||||||||||||||||||||||||||||||||||||||||||||||||
Sbjct  694  GTTACAGACCAGACAGTCGCCTTCGCCACTGGTGTTCCTCCAAATCTCTACGCATTTCAC  635

Query  182  CGCTACACTTGGAATTCCACTATCCTCTTCTGCACTCAAGTTCCCCAGTTTCCAATGACC  241
            ||||||||||||||||||||||||||||||||||||||||||||||||||||||||||||
Sbjct  634  CGCTACACTTGGAATTCCACTATCCTCTTCTGCACTCAAGTTCCCCAGTTTCCAATGACC  575

Query  242  CTCCACGGTTGAGCCGTGGGCTTTCACATCAGACTTAAGAAACCACCTGCGCGCGCTTTA  301
            ||||||||||||||||||||||||||||||||||||||||||||||||||||||||||||
Sbjct  574  CTCCACGGTTGAGCCGTGGGCTTTCACATCAGACTTAAGAAACCACCTGCGCGCGCTTTA  515

Query  302  CGCCCAATAAATCCGGACAACGCTTGCCACCTACGTATTACCGCGGCTGCTGGCACGTAG  361
            ||||||||||||||||||||||||||||||||||||||||||||||||||||||||||||
Sbjct  514  CGCCCAATAAATCCGGACAACGCTTGCCACCTACGTATTACCGCGGCTGCTGGCACGTAG  455

Query  362  TTAGCCGTGGCTTTCTAATAAGGTACCGTCAAGGTACGTTCATTTTCCTAACGTACTTGT  421
            ||||||||||||||||||||||||||||||||||||||||||||| ||||||||||||||
Sbjct  454  TTAGCCGTGGCTTTCTAATAAGGTACCGTCAAGGTACGTTCATTT-CCTAACGTACTTGT  396

Query  422  TCTTCCCTTACAACAGAGTTTTACGATCCGAAAACCTTCATCACTCACGCGGCGTTGCTC  481
            ||||||||||||||||||||||||||||||||||||||||||||||||||||||||||||
Sbjct  395  TCTTCCCTTACAACAGAGTTTTACGATCCGAAAACCTTCATCACTCACGCGGCGTTGCTC  336

Query  482  CATCAGACTTTCGTC-ATTGTGGAAGATTCC-TACTGCTGCCTCCCGTAGGAGTTTGGGC  539
            ||||||||||||||| ||||||||||||||| ||||||||||||||||||||||||||||
Sbjct  335  CATCAGACTTTCGTCCATTGTGGAAGATTCCCTACTGCTGCCTCCCGTAGGAGTTTGGGC  276

Query  540  CGTGTCTCAGTCCCAATGTGGCCGATCACCCTCTCAGGTCGGCTATGCATCGTTGCCTTG  599
            ||||||||||||||||||||||||||||||||||||||||||||||||||||||||||||
Sbjct  275  CGTGTCTCAGTCCCAATGTGGCCGATCACCCTCTCAGGTCGGCTATGCATCGTTGCCTTG  216

Query  600  GTAGGCCGTTACCCCCACCAACTAGCTAATGCACCGCGGGGCCCAT-CTACAGTGACGCC  658
            ||||||||||||||| |||||||||||||||||||||||| ||||| |||||||||||||
Sbjct  215  GTAGGCCGTTACCCC-ACCAACTAGCTAATGCACCGCGGG-CCCATCCTACAGTGACGCC  158

Query  659  GAAGCGCCTTTCAACTTCAAAACATGTGATTCGAAGGATTATCCGGG-TTAGCCCAGGTT  717
            ||||||||||||||||||||||||||||||||||||||||||||||  ||||||||||||
Sbjct  157  GAAGCGCCTTTCAACTTCAAAACATGTGATTCGAAGGATTATCCGGTATTAGCCCAGGTT  98

Query  718  TC  719
            ||
Sbjct  97   TC  96
```

Taxonomy

Reports

- **Lineage**

Organism	Blast Name	Score	Number of Hits	Description
Bacteria	bacteria		104	
.Kurthia	firmicutes		100	
..Kurthia gibsonii	firmicutes	1251	86	**Kurthia gibsonii hits**
..Kurthia sp.	firmicutes	1251	7	**Kurthia sp. hits**
..Kurthia sp. SCAU15	firmicutes	1251	1	**Kurthia sp. SCAU15 hits**
..uncultured Kurthia sp.	firmicutes	1251	1	**uncultured Kurthia sp. hits**
..Kurthia sp. B2	firmicutes	1251	1	**Kurthia sp. B2 hits**
..Kurthia sp. JB110_B12_VA_12EMRSA	firmicutes	1251	1	**Kurthia sp. JB110_B12_VA_12EMRSA hits**
..Kurthia sp. JB124_B12_VA_12EMRSA	firmicutes	1251	1	**Kurthia sp. JB124_B12_VA_12EMRSA hits**
..Kurthia sp. 11kri321	firmicutes	1251	1	**Kurthia sp. 11kri321 hits**
..Kurthia huakuii	firmicutes	1251	1	**Kurthia huakuii hits**

.bacterium peng-8	bacteria	1251	1	bacterium peng-8 hits
.bacterium peng-2	bacteria	1251	1	bacterium peng-2 hits
.uncultured bacterium	bacteria	1251	1	uncultured bacterium hits
.bacterium peng-9	bacteria	1251	1	bacterium peng-9 hits

- **Organism**

Description	Score	E value	Accession
Kurthia gibsonii [firmicutes]			
Kurthia gibsonii strain KG-01 16S ribosomal RNA gene, partial sequence	1251	0.0	PP389410
Kurthia gibsonii strain 1684 16S ribosomal RNA gene, partial sequence	1251	0.0	MW405846
Kurthia gibsonii strain 1717 16S ribosomal RNA gene, partial sequence	1251	0.0	MW405851
Kurthia gibsonii strain TB64A 16S ribosomal RNA gene, partial sequence	1251	0.0	PV686965
Kurthia gibsonii strain UN-06 16S ribosomal RNA gene, partial sequence	1251	0.0	OQ799123
Kurthia gibsonii strain T1G24 16S ribosomal RNA gene, partial sequence	1251	0.0	OQ472472
Kurthia gibsonii strain TB71 16S ribosomal RNA gene, partial sequence	1251	0.0	PV686972
Kurthia gibsonii strain IAE225 16S ribosomal RNA gene, partial sequence	1251	0.0	MK414929
Kurthia gibsonii strain NCB2 16S ribosomal RNA gene, partial sequence	1251	0.0	MK813166
Kurthia gibsonii strain 1716 16S ribosomal RNA gene, partial sequence	1251	0.0	MW405850
Kurthia gibsonii strain RCB894 16S ribosomal RNA gene, partial sequence	1251	0.0	KT261106
Kurthia gibsonii strain KAU-1 16S ribosomal RNA gene, partial sequence	1251	0.0	KT828545
Kurthia gibsonii strain Catla M14 16S ribosomal RNA gene, partial sequence	1251	0.0	ON491066
Kurthia gibsonii strain f1-fd1_D01.ab1 16S ribosomal RNA gene, partial sequence	1251	0.0	MW534484
Kurthia gibsonii strain NXGG_DHWF01 16S ribosomal RNA gene, partial sequence	1251	0.0	PV636459
Kurthia gibsonii strain CIFRI-AKSHG30 16S ribosomal RNA gene, partial sequence	1251	0.0	MW301114
Kurthia gibsonii strain AM031 16S ribosomal RNA gene, partial sequence	1251	0.0	OP755828
Kurthia gibsonii strain RCB321 16S ribosomal RNA gene, partial sequence	1251	0.0	KT260533
Kurthia gibsonii strain SAU_AFB01 16S ribosomal RNA gene, partial sequence	1251	0.0	MN658386
Kurthia gibsonii strain HBUAS71435 16S ribosomal RNA gene, partial sequence	1251	0.0	OR186313
Kurthia gibsonii strain Catla C15 16S ribosomal RNA gene, partial sequence	1251	0.0	ON491001
Kurthia gibsonii strain 48L4 16S ribosomal RNA gene, partial sequence	1251	0.0	MT192444
Kurthia gibsonii strain L10 16S ribosomal RNA gene, partial sequence	1251	0.0	KU315404
Kurthia gibsonii strain RCB309 16S ribosomal RNA gene, partial sequence	1251	0.0	KT260521
Kurthia gibsonii strain 1689 16S ribosomal RNA gene, partial sequence	1251	0.0	MW405849
Kurthia gibsonii strain TY-06 16S ribosomal RNA gene, partial sequence	1251	0.0	MN960342
Kurthia gibsonii strain HHR10 16S ribosomal RNA gene, partial sequence	1251	0.0	PP758843
Kurthia gibsonii strain TRM58103 16S ribosomal RNA gene, partial sequence	1251	0.0	PV383805
Kurthia gibsonii strain M25 16S ribosomal RNA gene, partial sequence	1251	0.0	OM617813
Kurthia gibsonii strain VCRI-NKL 16S ribosomal RNA gene, partial sequence	1251	0.0	PX397013
Kurthia gibsonii strain MF 16S ribosomal RNA gene, partial sequence	1251	0.0	KY606923
Kurthia gibsonii strain M15_4 16S ribosomal RNA gene, partial sequence	1251	0.0	PV653064
Kurthia gibsonii strain CEMTC_6271 16S ribosomal RNA gene, partial sequence	1251	0.0	PV436835
Kurthia gibsonii strain KG 03/24 16S ribosomal RNA gene, partial sequence	1251	0.0	PV147750
Kurthia gibsonii strain AM029 16S ribosomal RNA gene, partial sequence	1251	0.0	OP755827
Kurthia gibsonii strain HKG391 16S ribosomal RNA gene, partial sequence	1251	0.0	MT947182
Kurthia gibsonii strain KH2 16S ribosomal RNA gene, partial sequence	1251	0.0	PP178682
Kurthia gibsonii strain gol1 16S ribosomal RNA gene, partial sequence	1251	0.0	MK426815
Kurthia gibsonii strain Koac2 16S ribosomal RNA gene, partial sequence	1251	0.0	PQ236989
Kurthia gibsonii strain 1536 16S ribosomal RNA gene, partial sequence	1251	0.0	MW405841
Kurthia gibsonii strain AM007 16S ribosomal RNA gene, partial sequence	1251	0.0	OP755818
Kurthia gibsonii strain GI22 16S ribosomal RNA gene, partial sequence	1251	0.0	PQ524612
Kurthia gibsonii strain TB43 16S ribosomal RNA gene, partial sequence	1251	0.0	PV686944
Kurthia gibsonii strain ZZ12 16S ribosomal RNA gene, partial sequence	1251	0.0	KU234673
Kurthia gibsonii strain Molly7 16S ribosomal RNA gene, partial sequence	1251	0.0	OL454687
Kurthia gibsonii strain TB65 16S ribosomal RNA gene, partial sequence	1251	0.0	PV686966

Description	Score	E value	Accession
Kurthia gibsonii strain PHS2 16S ribosomal RNA gene, partial sequence	1251	0.0	ON858499
Kurthia gibsonii strain KH2 16S ribosomal RNA gene, partial sequence	1251	0.0	MN453416
Kurthia gibsonii strain ThlvkrLrS2 16S ribosomal RNA gene, partial sequence	1251	0.0	OR138056
Kurthia gibsonii strain beavul.ww1 16S ribosomal RNA gene, partial sequence	1251	0.0	OK138708
Kurthia gibsonii strain HQB1427 16S ribosomal RNA gene, partial sequence	1251	0.0	MH044656
Kurthia gibsonii strain NWMCC0045 16S ribosomal RNA gene, partial sequence	1251	0.0	MZ148523
Kurthia gibsonii strain MM10_3 16S ribosomal RNA gene, partial sequence	1251	0.0	PV653053
Kurthia gibsonii strain 1682 16S ribosomal RNA gene, partial sequence	1251	0.0	MW405844
Kurthia gibsonii strain PHN5 16S ribosomal RNA gene, partial sequence	1251	0.0	ON858497
Kurthia gibsonii strain NOORBSRD2 16S ribosomal RNA gene, partial sequence	1251	0.0	PP911484
Kurthia gibsonii strain M6 16S ribosomal RNA gene, partial sequence	1251	0.0	PP374613
Kurthia gibsonii strain BT15M 16S ribosomal RNA gene, partial sequence	1251	0.0	OL662824
Kurthia gibsonii strain Y15 16S ribosomal RNA gene, partial sequence	1251	0.0	OQ406180
Kurthia gibsonii strain B1 16S ribosomal RNA gene, partial sequence	1251	0.0	PQ849185
Kurthia gibsonii strain B1 16S ribosomal RNA gene, partial sequence	1251	0.0	PX884712
Kurthia gibsonii strain 1277 16S ribosomal RNA gene, partial sequence	1251	0.0	MW405836
Kurthia gibsonii strain TYL-A1 16S ribosomal RNA gene, partial sequence	1251	0.0	OP077323
Kurthia gibsonii strain 7C 16S ribosomal RNA gene, partial sequence	1251	0.0	MK104486
Kurthia gibsonii strain CBNU_45B1 16S ribosomal RNA gene, partial sequence	1251	0.0	PP598129
Kurthia gibsonii strain RMS4 16S ribosomal RNA gene, partial sequence	1251	0.0	KX950811
Kurthia gibsonii strain AM009 16S ribosomal RNA gene, partial sequence	1251	0.0	OP755819
Kurthia gibsonii strain Rohu T23 16S ribosomal RNA gene, partial sequence	1251	0.0	ON491161
Kurthia gibsonii strain B3 16S ribosomal RNA gene, partial sequence	1251	0.0	KM391941
Kurthia gibsonii strain IADCAMB12 16S ribosomal RNA gene, partial sequence	1251	0.0	MH619509
Kurthia gibsonii strain GUP 2 16S ribosomal RNA gene, partial sequence	1251	0.0	MK272939
Kurthia gibsonii strain AM013 16S ribosomal RNA gene, partial sequence	1251	0.0	OP755821
Kurthia gibsonii strain PHB2 16S ribosomal RNA gene, partial sequence	1251	0.0	ON858493
Kurthia gibsonii strain Catla C24 16S ribosomal RNA gene, partial sequence	1251	0.0	ON491019
Kurthia gibsonii strain TB62 16S ribosomal RNA gene, partial sequence	1251	0.0	PV686963
Kurthia gibsonii strain 1685 16S ribosomal RNA gene, partial sequence	1251	0.0	MW405847
Kurthia gibsonii strain Goldfish T11 16S ribosomal RNA gene, partial sequence	1251	0.0	ON491101
Kurthia gibsonii strain ND2 chromosome, complete genome	1251	0.0	CP147847
Kurthia gibsonii strain koi1 16S ribosomal RNA gene, partial sequence	1251	0.0	MK426822
Kurthia gibsonii strain SeqA3 16S ribosomal RNA gene, partial sequence	1251	0.0	MK294240
Kurthia gibsonii strain HBUAS82078 16S ribosomal RNA gene, partial sequence	1251	0.0	PV991465
Kurthia gibsonii strain CEMTC_7009 16S ribosomal RNA gene, partial sequence	1251	0.0	PV436836
Kurthia gibsonii strain TB70 16S ribosomal RNA gene, partial sequence	1251	0.0	PV686971
Kurthia gibsonii strain CIFRI.CH6 16S ribosomal RNA gene, partial sequence	1251	0.0	PV478494
Kurthia gibsonii strain AM001 16S ribosomal RNA gene, partial sequence	1251	0.0	OP755830
Kurthia gibsonii strain S3GA 16S ribosomal RNA gene, partial sequence	1245	0.0	OP824657
bacterium peng-8 [bacteria]			
Bacterium peng-8 16S ribosomal RNA gene, partial sequence	1251	0.0	KT698076
Kurthia sp. [firmicutes]			
Kurthia sp. strain Isolated_CD-F4Y 16S ribosomal RNA gene, partial sequence	1251	0.0	MW418170
Kurthia sp. strain BG15 16S ribosomal RNA gene, partial sequence	1251	0.0	OR975499
Kurthia sp. strain EB2016-75 16S ribosomal RNA gene, partial sequence	1251	0.0	MN367125
Kurthia sp. strain NwMCC01910045 16S ribosomal RNA gene, partial sequence	1251	0.0	MZ049633
Kurthia sp. strain NR1 16S ribosomal RNA gene, partial sequence	1251	0.0	MW036311
Kurthia sp. strain NR11 16S ribosomal RNA gene, partial sequence	1251	0.0	MW036326
Kurthia sp. strain NR19 16S ribosomal RNA gene, partial sequence	1251	0.0	MW036334
Kurthia sp. SCAU15 [firmicutes]			
Kurthia sp. SCAU15 16S ribosomal RNA gene, partial sequence	1251	0.0	KP125991

Description	Score	E value	Accession
uncultured Kurthia sp. [firmicutes]			
**Uncultured Kurthia sp. clone K-gibsonii-1 16S ribosomal RNA gene, partial sequence**	1251	0.0	**PQ865866**
bacterium peng-2 [bacteria]			
**Bacterium peng-2 16S ribosomal RNA gene, partial sequence**	1251	0.0	**KT698070**
Kurthia sp. B2 [firmicutes]			
**Kurthia sp. B2 16S ribosomal RNA gene, partial sequence**	1251	0.0	**KM391940**
uncultured bacterium [bacteria]			
**Uncultured bacterium clone JSP_7 16S ribosomal RNA gene, and 16S-23S ribosomal RNA intergenic spacer gene, region**	1251	0.0	**MH444929**
Kurthia sp. JB110_B12_VA_12EMRSA [firmicutes]			
**Kurthia sp. JB110_B12_VA_12EMRSA 16S ribosomal RNA gene, partial sequence**	1251	0.0	**KU644531**
Kurthia sp. JB124_B12_VA_12EMRSA [firmicutes]			
**Kurthia sp. JB124_B12_VA_12EMRSA 16S ribosomal RNA gene, partial sequence**	1251	0.0	**KU644530**
bacterium peng-9 [bacteria]			
**Bacterium peng-9 16S ribosomal RNA gene, partial sequence**	1251	0.0	**KT698077**
Kurthia sp. 11kri321 [firmicutes]			
**Kurthia sp. 11kri321 chromosome, complete genome**	1251	0.0	**CP013217**
Kurthia huakuii [firmicutes]			
**Kurthia huakuii strain FPM-CIP-1 16S ribosomal RNA gene, partial sequence**	1251	0.0	**KY616642**

- ## Taxonomy

Taxonomy	Number of hits	Number of Organisms	Description
Bacteria	104	13	
. Kurthia	100	9	
.. Kurthia gibsonii	86	1	**Kurthia gibsonii hits**
.. unclassified Kurthia	12	6	
... Kurthia sp.	7	1	**Kurthia sp. hits**
... Kurthia sp. SCAU15	1	1	**Kurthia sp. SCAU15 hits**
... Kurthia sp. B2	1	1	**Kurthia sp. B2 hits**
... Kurthia sp. JB110_B12_VA_12EMRSA	1	1	**Kurthia sp. JB110_B12_VA_12EMRSA hits**
... Kurthia sp. JB124_B12_VA_12EMRSA	1	1	**Kurthia sp. JB124_B12_VA_12EMRSA hits**
... Kurthia sp. 11kri321	1	1	**Kurthia sp. 11kri321 hits**
.. uncultured Kurthia sp.	1	1	**uncultured Kurthia sp. hits**
.. Kurthia huakuii	1	1	**Kurthia huakuii hits**
. unclassified Bacteria	3	3	
.. bacterium peng-8	1	1	**bacterium peng-8 hits**
.. bacterium peng-2	1	1	**bacterium peng-2 hits**
.. bacterium peng-9	1	1	**bacterium peng-9 hits**
. uncultured bacterium	1	1	**uncultured bacterium hits**

Top
Follow NCBI
